# Electroacupuncture improves cognitive function and neuropsychiatric symptoms in breast cancer survivors: a pilot randomized controlled trial

**DOI:** 10.1093/jnci/djag096

**Published:** 2026-09-01

**Authors:** Ding Quan Ng, Matthew Heshmatipour, Julia Trudeau, Apeksha Sridhar, Brock Pluimer, Olivia G.G. Drayson, Sayeh M. Lavasani, Ritesh Parajuli, Sanghoon Lee, Anshu Agrawal, Munjal M. Acharya, Charles L. Limoli, Richard E. Harris, Lifang Xie, Shaista Malik, Alexandre Chan

**Affiliations:** 1 Department of Clinical Pharmacy Practice, School of Pharmacy and Pharmaceutical Sciences, University of California, Irvine, CA, United States; 2 Susan Samueli Integrative Health Institute, University of California Irvine, Irvine, CA, United States; 3 Department of Radiation Oncology, School of Medicine, University of California, Irvine, CA, United States; 4 Division of Hematology/Oncology, Department of Medicine, School of Medicine, University of California, Irvine, CA, United States; 5 College of Korean Medicine, Kyung Hee University, Seoul, South Korea; 6 Division of Basic and Clinical Immunology, Department of Medicine, School of Medicine, University of California, Irvine, CA, United States; 7 Department of Anatomy & Neurobiology, School of Medicine, University of California, Irvine, CA, United States; 8 Department of Anesthesiology and Perioperative Care, School of Medicine, University of California, Irvine, CA, United States

## Abstract

**Background:**

We conducted a randomized, double-blinded pilot trial to compare the impact of 2 electroacupuncture (EA) regimens on co-occurring neuropsychiatric symptoms among breast cancer survivors.

**Methods:**

Breast cancer survivors (BCS) who self-reported cognitive impairment, fatigue, insomnia, or psychological distress were randomized (1:1) to receive 10 weekly EA to target either neuropsychiatric-specific (neuropsychiatric-specific EA, nEA) or nonneuropsychiatric-specific (sham EA, sEA) acupoints. Primary endpoints were the within-group pre-post effect sizes (Glass’s Δ) in symptom severities, adjusted for multiple comparisons (*P*_adjusted_). Outcomes were assessed using neurocognitive tests (CANTAB), patient-reported outcomes (PROs) (Functional Assessment of Cancer Therapy-Cognitive Function, Multidimensional Fatigue Symptom Inventory-Short Form, European Organisation for Research and Treatment of Cancer Quality of Life Questionnaire-Core 30), plasma biomarkers, and neuroimaging. Responders were defined by reliable change index (for objective cognition) or minimal clinically important differences (for PROs).

**Results:**

Thirty-five participants were recruited, with 30 (86%) completing all sessions. The mean (±SD) age was 58.2 (±12.2) years, and 86% reported co-occurring symptoms. Following treatment, the nEA group demonstrated significant improvements in attention (T3: Δ = 0.562, T4: Δ = 0.708, both *P*_adjusted_ <.05) and distress (T3: Δ = 0.764, T4: Δ = 0.711, both *P*_adjusted_ < .05). More responders were observed after nEA treatment for objective cognition (42.9% vs 12.5%) and distress (50% vs 37.5%). Neuropsychiatric-specific EA-treated participants showed increased gray matter volume compared with sEA (*P* = .033), which positively correlated with better attention function (*r* = 0.69, *P* = .020). Neuropsychiatric-specific EA-related improvements in memory and response speed were associated with reduced connectivity in the default mode network (DMN-SFG, *r* = −0.93, *P* < .01) and increased connectivity in the dorsal attention network (DAN-SMG, *r* = 0.86, *P* < .001), respectively. All adverse events were grade 2 or lower.

**Conclusion(s):**

Electroacupuncture targeting neuropsychiatric-specific acupoints suggests improvements in cognition and distress symptoms in BCS, warranting validation in larger, multicenter trials.

**Clinicaltrials.gov:**

NCT05283577.

## Background

Advances in breast cancer screening and treatment have significantly elevated the 5-year relative survival rate above 90%, with more than 4 million women living in United States with a history of breast cancer.^1,2^ In support of this burgeoning population, we are seeing a growing emphasis on enhancing cancer survivorship to address the lack of infrastructure and services to manage a range of neuropsychiatric symptoms (ie, cognitive impairment, fatigue, distress, and insomnia) experienced by patients during and after cancer treatment.^1,3,4^ Patients have expressed that this lack of support exacerbated emotional distress, difficulties in navigating survivorship and postcancer recovery, and a sense of abandonment by the institution.^5^ Concurrently, prescribing multiple psychotropic medications for co-occurring symptoms presents a significant challenge in oncology.^6^ Clinicians must carefully weigh the benefits against the risks of polypharmacy, including heightened risks of adverse reactions, drug-drug interactions with systemic therapies, and long-term implications of medication dependence during cancer survivorship.^7,8^ Effective, safe nonpharmacological strategies are needed to manage a variety of neuropsychiatric symptoms in breast cancer.

Growing evidence supports acupuncture as a modality to manage cancer- and associated treatment-related physical and mental health symptoms.^9,10^ Electroacupuncture (EA) enhances manual acupuncture by applying a small electrical current to the needles.^11^ A number of acupoints have been shown to improve neuropsychiatric symptoms. ST36 *Zusanli* induces anti-inflammatory and neuromodulating effects,^12-14^ and is often used for fatigue and general wellbeing.^15^ GV20 *Baihui*, HT7 *Shenmen*, and SP6 *Sanyinjiao* have been shown to alleviate insomnia, distress, and cognitive impairment via the upregulation of brain-derived neurotrophic factor (BDNF), a key neurotrophic factor for the proliferation and maintenance of neuronal precursors and neurons.^16-21^ Both decreased neurogenesis and increased inflammation are implicated in the pathogenesis of neuropsychiatric symptoms among breast cancer survivors.^22-24^

Despite guidelines recommending acupuncture in breast cancer survivorship,^9^ further evidence is needed on the benefit of disease-specific acupoint regimens. This pilot randomized controlled trial (RCT) evaluated the preliminary efficacy and safety of 2 different EA regimens administered at neuropsychiatric-specific (neuropsychiatric-specific EA, nEA) vs nonneuropsychiatric-specific (sham EA, sEA) acupoints on cognition, fatigue, distress, and insomnia. Our use of an active control (sEA) with electrical stimulation but varied needle placement allows us to isolate the specific therapeutic effect of acupoint selection. Our strategy incorporated patient-reported outcomes (PROs) alongside objective surrogate markers of inflammation, neuronal health, and brain function to provide an impartial and mechanistic basis for the observed clinical effects.

## Methods

### Trial design

We conducted a randomized (1:1), controlled, patient and assessor-blinded pilot trial at the Chao Family Comprehensive Cancer Center and the Susan Samueli Integrative Health Institute from 2022 to 2024 (ClinicalTrials.gov: NCT05283577). We received ethics approval from the UC Irvine Institutional Review Board (#2021-6732). Our recruitment target was 34 participants, with 30 evaluable participants (15 in each arm, assumed dropout of 10%) following published guidance for pilot trials.^25-28^ The study protocol has been peer-reviewed and published^29^; a detailed description of the methods can be found in Supplementary Methods.

### Participant eligibility

Inclusion criteria were: ≥18 years old, diagnosed with cancer, received anticancer treatment, life expectancy of ≥6 months, and reporting ≥1 of the neuropsychiatric symptom(s) (ie, cognitive impairment, fatigue, insomnia, anxiety or depression) over the past 7 days.

Exclusion criteria were: severe needle phobia, severe psychiatric/mental disorders affecting neurocognitive assessment, bleeding disorders, pacemakers/electronic implants, epilepsy, acupuncture in the prior 3 months, or being pregnant/planning pregnancy/breastfeeding.

Patients with magnetic metal implants, severe claustrophobia, or nonremovable metallic dental devices were ineligible for neuroimaging but could still receive EA interventions.

### Intervention

Trained, licensed acupuncturists conducted weekly EA treatments for 10 weeks.^29^ Participants were supine while needles were inserted 9-24 mm, at an insertion angle of 15-90° depending on the usual technique for a given acupoint. Electrodes from the EA unit ES-160 (Ito Co. Ltd) were attached to the needle handles, providing 30 min of 2 Hz continuous wave EA stimulation with personalized intensity.

- *nEA:* Participants received EA at disease-related acupoints selected by an experienced licensed acupuncturist. Acupoint selection was further rationalized by published scientific and clinical evidence (Table S1).
- *sEA:* Participants received EA at nondisease-related acupoints using stimulation and intensity parameters indistinguishable from the nEA group to maintain blinding.

### Data collection

**Figure 1.**
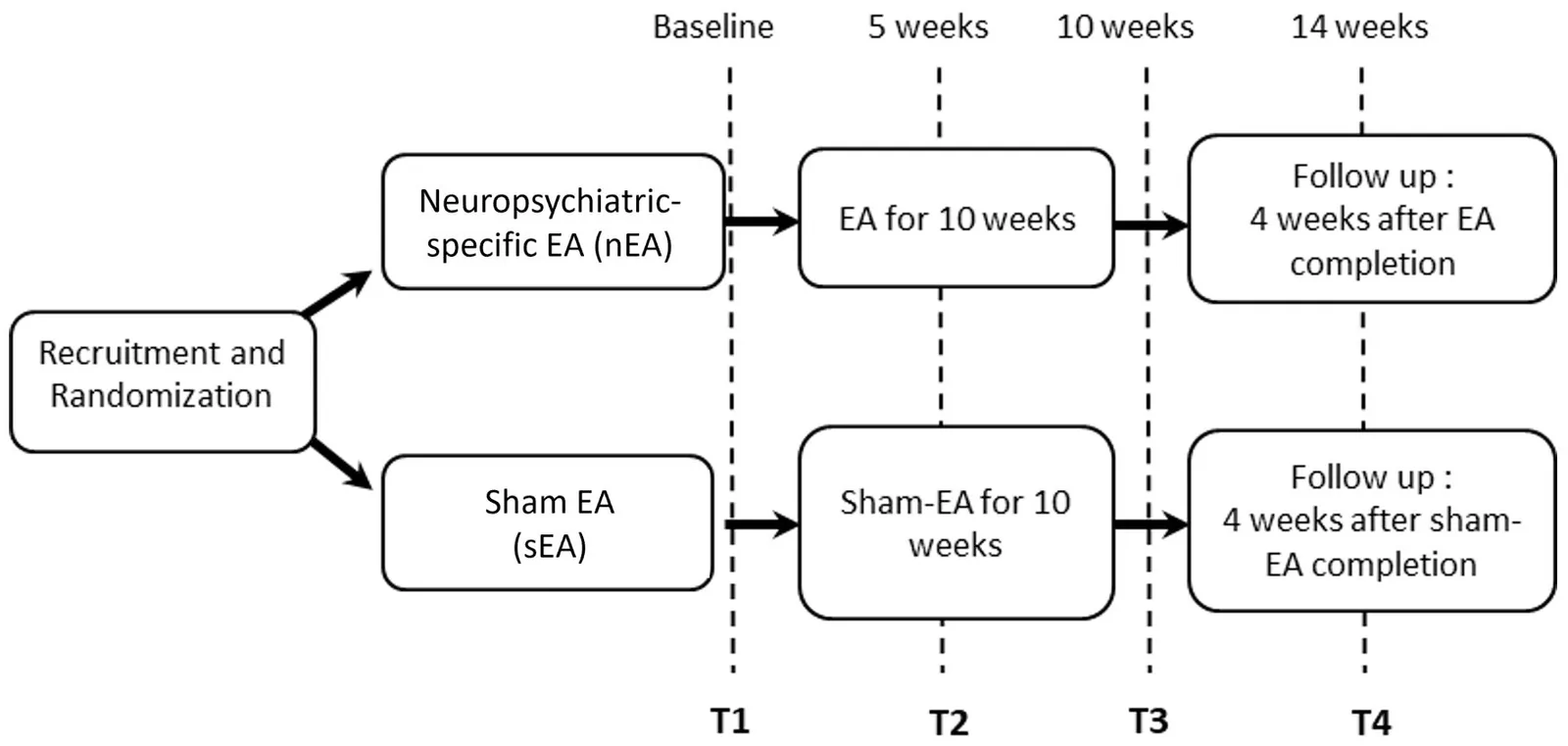
Study assessment time points (T1 denotes time point 1, T2 time point 2 and so on). The duration of this study is approximately 14 weeks, composed of a 10-week treatment period and a study completion visit at 14 weeks.

Patients were assessed at 4 timepoints: baseline (T1), midtreatment (T2, ~5 weeks after baseline), end-of-treatment (T3, ~10 weeks after baseline), and ~4 weeks after treatment (T4) (Figure 1). Baseline characteristics were collected at T1. Participants completed a cognitive test battery and PRO questionnaires, and provided a 10-mL peripheral blood sample at all time points. Neuroimaging was performed at T1 and T3 for eligible patients, and a feedback survey was administered posttreatment.

### Objective cognition

Objective cognition was assessed using the Cambridge Neuropsychological Test Automated Battery (CANTAB), a computerized cognitive test battery, administered on a tablet. Following International Cognition and Cancer Task Force (ICCTF) guidance, we evaluated cognitive performance across 5 domains: attention, memory, response speed, executive function, and multitasking^30,31^ (Table S2).

### Patient-reported outcomes

Several PROs were utilized to assess patients’ self-reported symptoms. The Functional Assessment of Cancer Therapy-Cognitive Function (FACT-Cog) version 3 measured self-perceived cognition.^32,33^ Fatigue symptoms were captured using the Multidimensional Fatigue Symptom Inventory-Short Form (MFSI-SF) questionnaire.^34,35^ The European Organisation for Research and Treatment of Cancer Quality of Life Questionnaire-Core 30 (EORTC QLQ-C30) was used to assess psychological distress (Emotional Function [EF] subscale), insomnia (SL subscale), and overall quality-of-life (QoL, via Global Health Status [GHS] subscale).^36,37^

### Plasma biomarkers

Cytokines assessed included chemokine (C-X-C motif) ligand 13 (CXCL13), interleukin (IL)-1α, IL-1β, IL-4, IL-6, IL-8, IL-10, IL-17A, IL-18, IL-21, IL-23, IL-33, monocyte chemoattractant protein-1 (MCP-1), macrophage inflammatory protein-1 alpha (MIP-1α), RANTES (regulated on activation, normal T-cell expressed and secreted, ie, chemokine [C-C motif] ligand 5), and tumor necrosis factor alpha (TNF-α). Tropomyosin receptor kinase B (TrkB) and cytokine levels were quantified using ProcartaPlex immunoassays (Thermo Fisher Scientific). BDNF levels were measured using a commercially available enzyme-linked immunosorbent assay kit (Biosensis). Concentrations were determined from standard curves and presented in picograms per milliliter (pg/mL). Undetectable levels based on assay sensitivity were set to 0pg/mL. Biomarkers with >80% of values that were below the lower limit of quantification were excluded from the final analysis.^22^

### Neuroimaging procedures

All neuroimaging procedures were completed using a 3 T Siemens Prisma scanner with a 32-channel head coil at the UC Irvine Facility for Imaging and Brain Research. Following ICCTF’s recommendations, we performed resting-state functional MRI (fMRI), T1- and T2-weighted sequences as per the Alzheimer’s Disease Neuroimaging Initiative 3 MRI protocol.^38^

### Safety, patient acceptance and blinding assessment

Adverse events (AEs) were graded according to the Common Terminology Criteria for Adverse Events version 5.0.^39^ After completing all EA sessions, participants were asked to guess their group allocation (treatment, control, or “not sure”) and whether they were satisfied, felt they benefited, and would consider EA again outside of a trial.

### Study endpoints

To evaluate preliminary efficacy of the regimen, we utilize coprimary endpoints comprising within-group changes from baseline to end-of-treatment (T3) and posttreatment (T4) across 10 independent symptom domains encompassing objective cognitive domains (attention, memory, response speed, executive function, multitasking) and patient-reported outcomes (subjective cognition, fatigue, distress, insomnia, quality of life). To maintain the integrity of the findings, we applied the Benjamini-Hochberg method to account for multiple testing across the symptom domains at each timepoint.

Secondary endpoints evaluated between-group changes, compared proportions of treatment responders, and compared the proportions of AEs between nEA and sEA. Responders for PROs were defined as symptom improvement that had achieved minimal clinically important differences (MCID),^35,40-43^ and for objective cognition by achieving clinically significant improvement in at least 1 cognitive domain(s), achieving reliable change index (>1.96) at T3 and T4.^44,45^ Calculated between-group effect sizes will inform future sample size requirements for a future, larger scale definitive RCT.

Exploratory endpoints included the pre-post changes in plasma and neuroimaging biomarkers, and their correlations with health outcomes, pre-post changes in health utility, safety, and feasibility.

### Sample size calculation

As this is a preliminary efficacy pilot and existing literature lacks precise variance estimates for the differences in neuropsychiatric outcomes between nEA and sEA, sample size for this study was not determined based on a formal power calculation for hypothesis testing. Instead, the study aims to provide the necessary parameters to inform a future definitive trial. Following published guidance for sample size recommendations in pilot trials,^25-28^ we recruited a minimum of 30 evaluable participants completing all research procedures, with 15 in each arm. Our final sample size, after accounting for an assumed dropout rate of 10%, is 34 participants.

### Statistical analysis

For the primary endpoint, we employed linear mixed models with fixed effects (baseline outcome, group, time, group-time interaction) and random intercepts for participants, using a modified intention-to-treat approach. Linear mixed models were used to compute within-group, pre-post effect sizes (Glass’s Δ; small [0.2], medium [0.5], large [0.8]^46,47^) adjusted for baseline. Multiple testing of pre-post changes was controlled using the Benjamini-Hochberg method, and statistical significance was defined as an adjusted *P* value (*P*_adjusted_) of <.05.^48^ A sensitivity analysis adjusted for the placebo effect by including participants’ treatment guesses as a confounder. Between-group effect sizes were presented using Cohen’s *d* (positive values favor nEA). Proportions of treatment responders (defined separately for each health outcome and for the combined summation of all outcomes) were descriptively summarized for comparison between the groups.

Plasma biomarker values were normalized (natural log transformation) and standardized (*z*-scores) before modeling. Undetectable values were imputed as 0.001. Linear mixed models with the same fixed effects, plus a random slope for batch-to-batch variability, were used to model postintervention minus baseline changes. We used Spearman correlation to examine the relationship between relative changes (baseline to T3 or T4) in plasma biomarkers and standardized health outcomes (where positive values indicate improvement).

All analyses were 2-tailed, tested at 5% significance level, and performed with R v4.4.1^49^ unless otherwise specified.

### Neuroimaging analysis

Structural T1- and T2-weighted MRI images were analyzed using FSL (FMIRB Software Library)^50^ to calculate gray matter and white matter volumes, with hippocampal volume determined separately via the FIRST tool. Neurocognitive scores were correlated with these metrics using Pearson’s analysis within each treatment group.

Functional MRI data were preprocessed using fMRIPrep (v23.2.0).^51^ Resting-state networks were identified using group-level independent component analysis (ICA) via FSL’s MELODIC. Spatial maps were matched to canonical templates^52,53^ to identify the default mode (DMN), sensorimotor (SMN), frontoparietal (FPN), and dorsal attention (DAN) networks, as well the left and right insular cortex (IC) from the Harvard-Oxford atlas.

Functional connectivity (FC) analyses were conducted using the CONN toolbox. Seed-to-whole-brain analyses used the ICA-derived networks and the IC seeds. Second-level analysis of FC changes modeled group differences using mean-centered changes in objective cognition (memory, attention, response speed) as covariates, due to observed nEA-specific improvements. Outliers and volumes with excessive head motion were excluded. Statistical significance was set at a voxelwise *P <*.001 (uncorrected), with cluster-level familywise error correction at *P* <.05.

For neuroimaging-outcomes correlation analyses, health outcomes were standardized so that higher scores represented better outcomes.

## Results

### Participant characteristics

**Figure 2.**
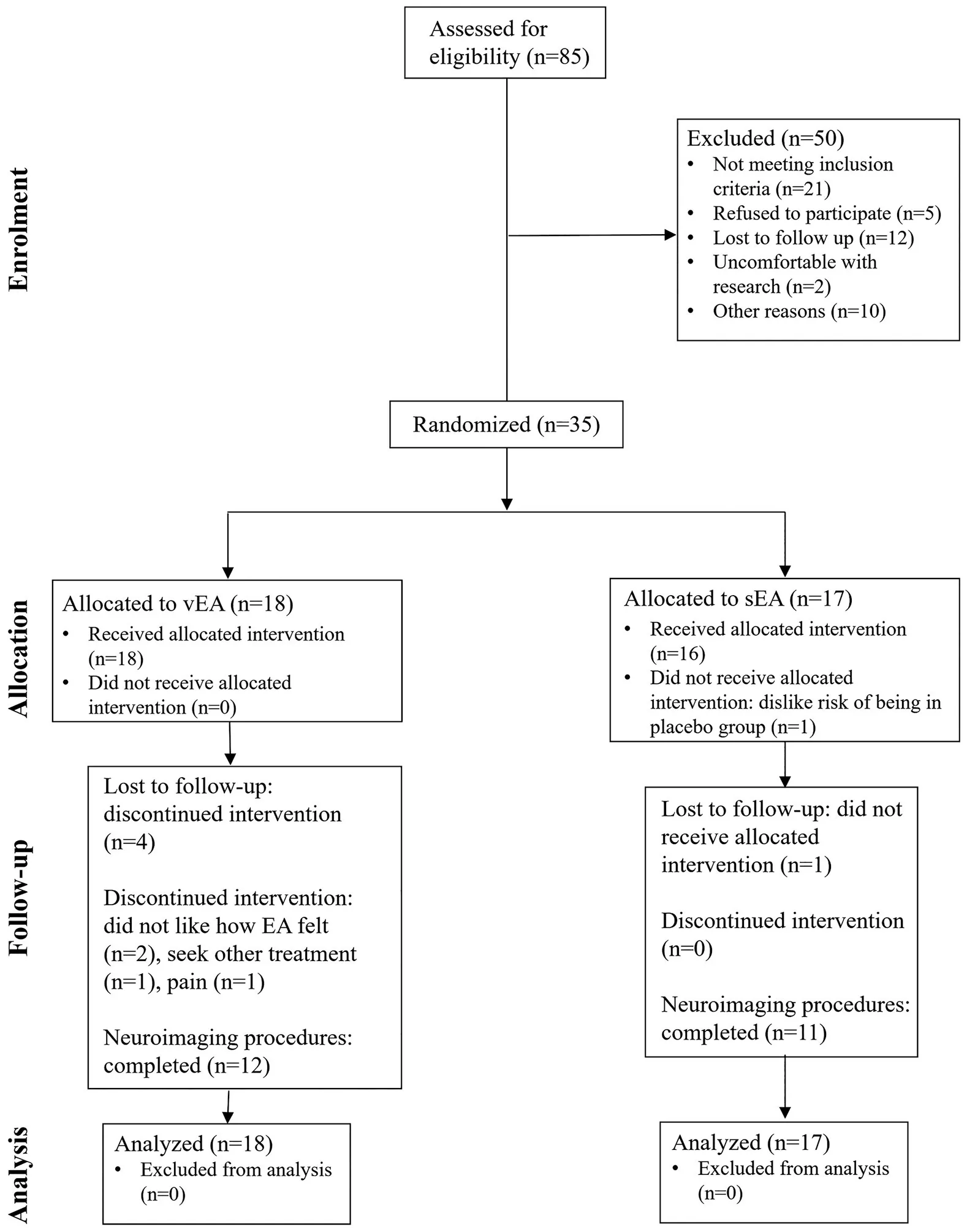
CONSORT flow diagram. All observations were included in relevant analyses following modified intention-to-treat principles for the primary endpoint analysis.

We recruited 35 participants (mean age = 58.2), with 5 lost to follow-up, leaving 30 (85.7%) completing all treatment sessions. Eighteen received nEA and 17 received sEA. Four participants dropped out before the T2 assessment, and one before T3 (Figure 2). Sociodemographic and cancer treatment-related characteristics were comparable except for age at diagnosis and education level (Table 1).

### Greater objective cognitive improvement in the nEA group

**Figure 3.**
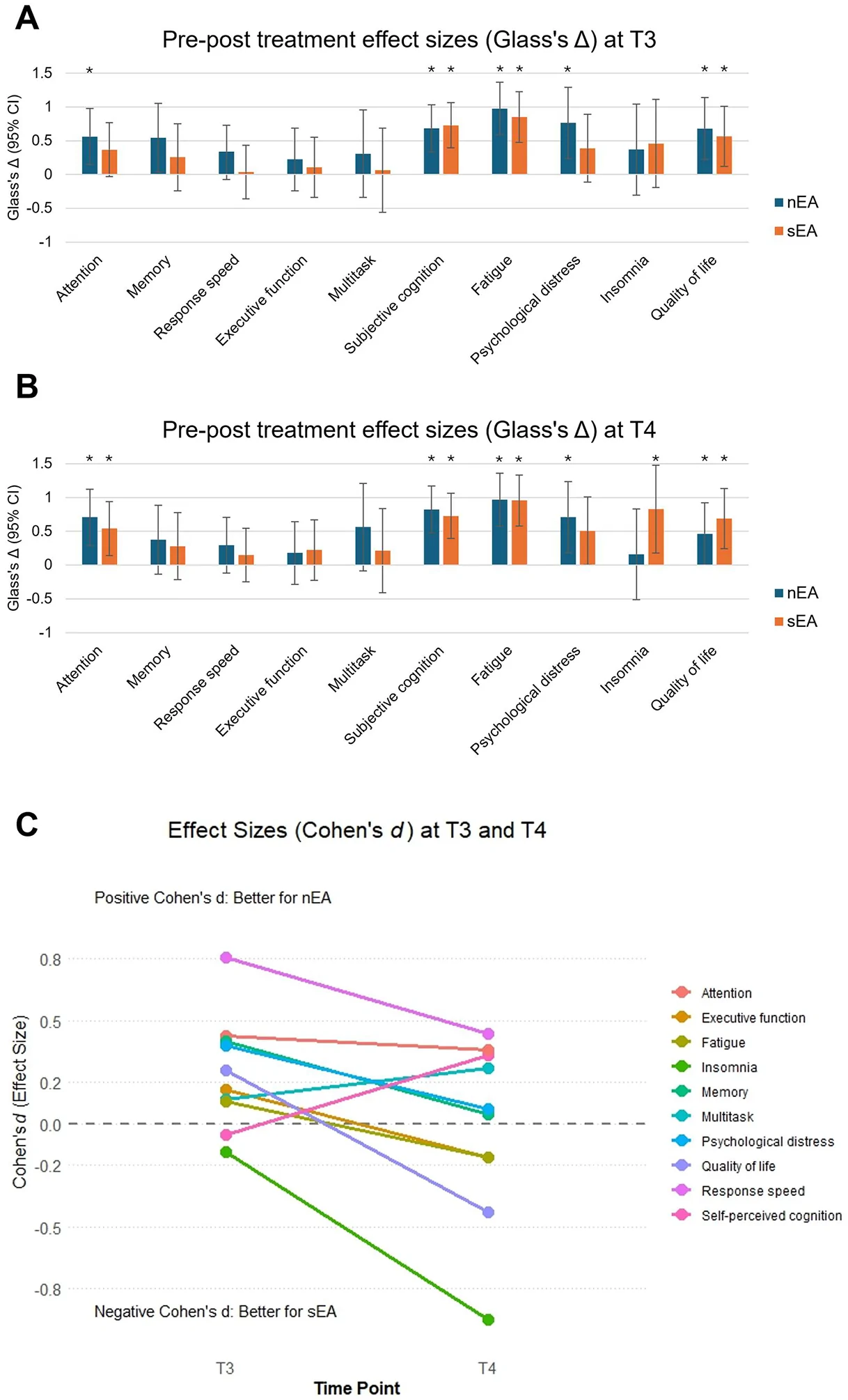
Comparison of health outcomes for neuropsychiatric-specific electroacupuncture (nEA) vs sham electroacupuncture (sEA) after treatment (T3) and at follow-up (T4). **A, B)** Bar graphs showing baseline-adjusted, treatment-specific pre-post effect sizes (Glass’s Δ with 95% CI) of health outcomes estimated from linear mixed models. Positive values for Glass’s Δ (small [0.2], medium [0.5], large [0.8]) indicate improvements from pretreatment levels. **A, B)** *P_adjusted_ <.05 (Benjamini-Hochberg method for multiple testing correction). **C)** Dot and line plots display Cohen’s *d* effect sizes relative to sEA. Positive values for Cohen’s *d* (small [0.2], medium [0.5], large [0.8]) indicate greater improvements in nEA compared with sEA.

Health outcomes are summarized in Table S3. After correcting our primary endpoint with multiple testing, both nEA and sEA treatment significantly improved in self-perceived cognition (nEA: Δ = 0.685; sEA: Δ = 0.730, both *P*_adjusted_ <.05) and fatigue (nEA: Δ = 0.978; sEA: Δ = 0.853, both *P*_adjusted_ <.05) after treatment (T3, Figure 3A) and remained durable 4 weeks later (T4, Figure 3B). Further analysis of specific primary endpoints revealed distinct, regimen-specific improvements. Neuropsychiatric-specific EA demonstrated significant pre-post improvement in attention (nEA, T3: Δ = 0.562, T4: Δ = 0.708, both *P*_adjusted_ <.05) and distress (nEA, T3: Δ = 0.764, T4: Δ = 0.711, both *P*_adjusted_ <.05), while sEA showed improvement in QoL (sEA, T3: Δ = 0.568, T4: Δ = 0.686, both *P*_adjusted_ <.05). All within-group changes are reported in Table S4.

At T3, the largest improvements were seen in response speed, with a large effect size (*d* = 0.805, *P* >.05) trending to favor nEA (Figure 3). At T4, the largest difference favored the sEA group for insomnia (*d* =−0.950, *P* <.05) (Figure 3C). Findings remain similar after adjusting treatment guesses (Table S5) and also by age at diagnosis and education (Table S6).

At T3, more than 80% of both arms reported improvement in at least one symptom. The largest difference was observed in objective cognition (nEA = 42.9%; sEA = 12.5%; *P* = .101) and psychological distress (nEA = 50%; sEA = 37.5%; *P*=.713) favoring nEA participants. In contrast, a larger difference favoring the sEA arm was observed with self-perceived cognition (nEA = 42.9%; sEA = 56.3%; *P*=.715). These responses were maintained at 4 weeks posttreatment (T4), with the largest difference favoring the nEA arm in objective cognition (nEA = 35.7%; sEA = 12.5%; *P*=.204) and psychological distress (nEA = 50%; sEA = 31.3%; *P*=.457) (Table S7).

### Inflammatory marker reductions were associated with symptoms improvement

Interleukin-1β, IL-4, and IL-23 were excluded from the analysis as >80% of their values were below the lower limit of quantification. A descriptive summary can be found in Table S8.

In the nEA arm, a medium reduction of IL-8 was observed (Δ = −0.593; *d* = −1.132) by the end of the treatment. At 4 weeks posttreatment, a medium increase over time was observed with IL-33 (Δ = 0.506; *d* = 1.341) (Table S9). Within the nEA group, changes in health outcomes were significantly correlated with biomarkers including BDNF (with fatigue, *rho* = −0.65), TrkB (with attention, *rho* = −0.62), CXCL13 (with attention, *rho* = −0.66), IL-10 (with response speed, *rho* = 0.66), MCP-1 (with multitasking, *rho* = 0.6), and TNF-α (with self-perceived cognition, *rho* = −0.64).

In the sEA arm, a medium increase of BDNF was observed by the end of the treatment (Glass’s Δ = 0.511; *d* = 0.589) (Table S9). Biomarkers significantly correlated with health outcomes included BDNF (with insomnia, *rho* = 0.6), TrkB (with response speed, *rho* = 0.51), IL-1α (with response speed, *rho* = −0.62), IL-6 (with QoL, *rho* = −0.56), IL-10 (with fatigue, *rho* = −0.5), IL-17A (with insomnia, *rho* = 0.57), IL-18 (with response speed, *rho* = −0.54), IL-21 (with response speed, *rho* = −0.53), MIP-1α (with response speed, *rho* = −0.53), RANTES (with response speed, *rho* = −0.64; with insomnia, *rho* = 0.75), and TNF-α (with response speed, *rho* = −0.54).

All pairwise correlations are presented in Figure S1.

### Brain structural changes observed in the nEA group

**Figure 4.**
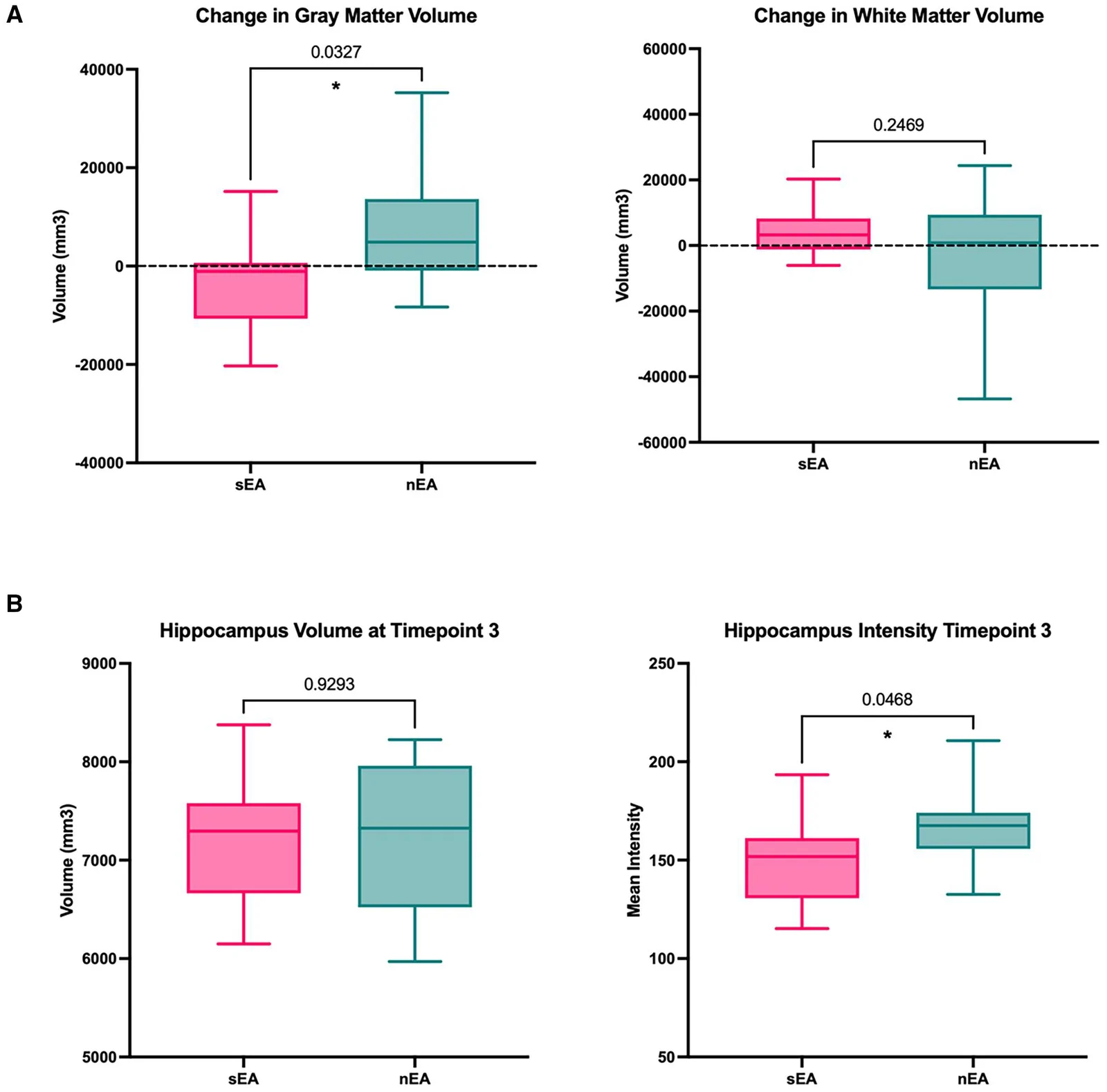
Comparison of structural image metrics between neuropsychiatric-specific electroacupuncture (nEA) and sham electroacupuncture (sEA). **A)** Changes in gray matter and white matter volume between T1 (baseline) and T3 (after treatment completion), stratified by treatment group. **B)** Hippocampal volume and mean intensity at T3, stratified by treatment group. *P <.05.

A significant difference between the treatment groups was observed in the change in total gray matter volume (*P* =.033) (Figure 4), where in the sEA group we observed a significant reduction in gray matter volume compared with the nEA group. The change in white matter was not significant or statistically different between the groups (*P* =.247). No significant changes in hippocampal volume were observed but at the late timepoint, the mean T1-weighted intensity across the hippocampi was significantly greater in the nEA group than the sEA group (*P* =.047).

Correlation analysis at T3 revealed that favorable clinical outcomes in the nEA group associated with structural changes: better attention correlated with increased gray matter volume (*r* = 0.69, *P*=.020), higher QoL with greater hippocampal intensity (*r* = 0.66, *P* =.026), and improved executive function with greater hippocampal volume (*r* = 0.68, *P* =.020) (Table S10).

### Brain connectivity was linked to cognitive improvement in the nEA group

**Figure 5.**
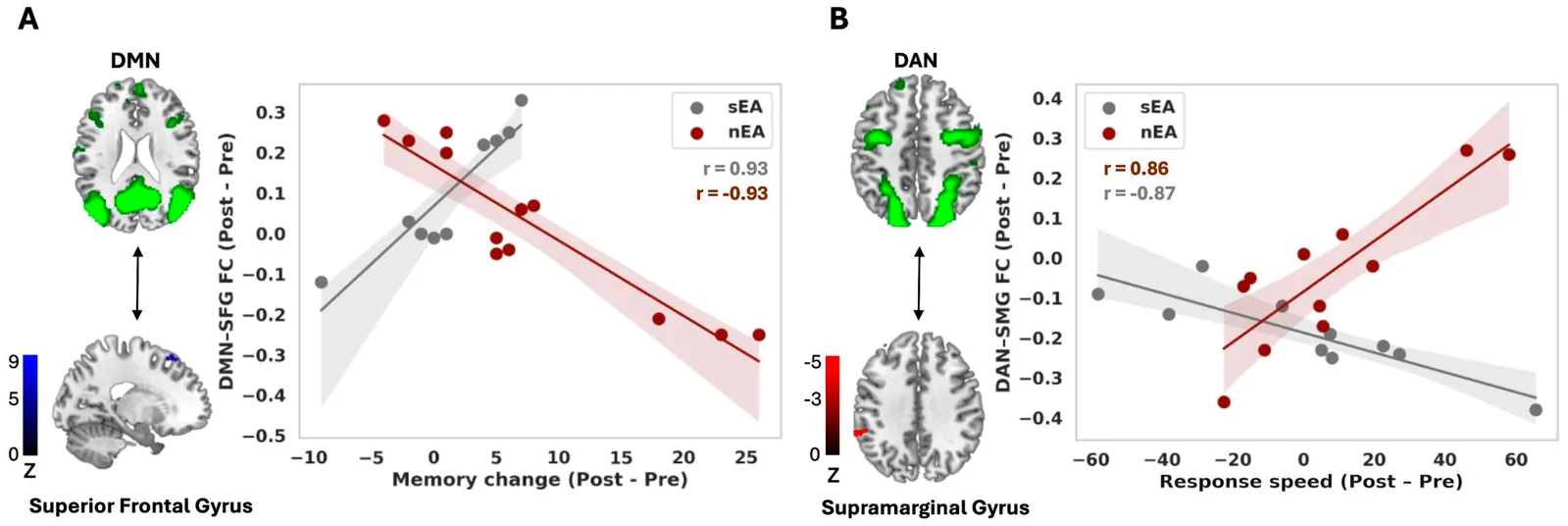
Functional connectivity changes associated with cognitive improvement following treatments. **A)** Scatterplot showing the association between changes in functional connectivity (FC) between the default mode network (DMN) and superior frontal gyrus (SFG) and changes in memory performance (post-pre). A negative correlation was observed in the nEA group (maroon; *r*=−0.93) and a positive correlation in the sEA group (gray; *r* = 0.93). Brain maps show the DMN seed (top) and SFG cluster (bottom). **B)** Scatterplot showing the association between changes in FC between the dorsal attention network (DAN) and supramarginal gyrus (SMG) and changes in response speed (post-pre). A positive correlation was observed in the nEA group (*r*=0.86) and a negative correlation in the sEA group (*r*=−0.87). Lower response speed scores indicate faster performance. Brain maps show the DAN seed (top) and SMG cluster (bottom). **A, B)** Shaded areas represent 95% CIs. Both memory and response speed changes were standardized so that higher scores represent improved outcomes.

Between-group analyses revealed distinct patterns in FC-cognition relationships, particularly for memory and response speed. In the nEA group, reduced DMN-left superior frontal gyrus (SFG) connectivity was associated with greater memory improvement (*r* = −0.93, *P* <.001, Figure 5A), whereas increased DAN-left primary somatosensory cortex (S1) and right FPN-inferior frontal gyrus (IFG) connectivity was also linked with greater memory gains (*r* = 0.79, *P* =.002; *r* = 0.57, *P* =.05, respectively). In contrast, the sEA group demonstrated the opposite pattern, such that memory improvement was related to increased DMN-left SFG connectivity (*r* = 0.93, *P <*.001, Figure 5A) but reduced DAN-left S1 connectivity and right FPN-IFG connectivity (*r* =−0.90, *P* =.001; *r*=−0.91, *P* =.001, respectively).

For response speed, faster response speed was associated with increased connectivity between the DAN-left supramarginal gyrus (SMG) and right FPN-SMG/S1 (*r* = 0.86, *P <*.001; *r*= 0.84, *P* =.001, respectively) in the nEA group, whereas the sEA group showed the opposite pattern, with faster response speed associated with reduced FC between the same regions (*r*=−0.87, *P* =.001; *r*=−0.80, *P* =.005, respectively) (Figure 5B).

Representative examples of these patterns are illustrated in Figure 5, showing FC changes related to improvements in memory (DMN-SFG) and response speed (DAN-SMG) following nEA. Details of all significant clusters are presented in Table S11.

### Few adverse effects observed

The majority of AEs were grade 1, with grade 2 pain reported in 1 (5.6%) participant who received nEA treatment. The breakdown of grade 1 AEs was as follows: nEA participants reported bleeding (*n* = 1, 5.6%), and sEA participants recorded numbness (*n*= 2, 12.5%), bruising (*n* = 2, 12.5%), nausea (*n* = 1, 6.3%), and redness (*n* =1, 6.3%).

### Favorable perceptions toward EA

More than 80% of participants in this study, regardless of treatment allocation, reported favorable perceptions toward participation in an EA trial and intervention. Neuropsychiatric-specific EA patients were more likely (50%) to guess correctly on their treatment allocation compared with sEA (37.5%). Key reasons for participant satisfaction with the trial were the relaxing experience (*n* = 18, 60%) and perceived symptom improvement (*n* = 5, 16.7%) and adjacent health benefits such as pain relief (*n* = 3, 10%) derived from the EA treatment (Table S12).

## Discussion

In this randomized, controlled, patient and assessor-blinded pilot trial, we found that patients treated with a regimen consisting of acupoints targeting nEA showed significantly greater immediate improvements in objective cognition and psychological distress than in the sEA control group (T3), with the cognitive improvements proving more durable at the 4-week follow-up (T4). Between-group differences in fatigue and insomnia were modest at T3. Notably, the sEA group continued to improve in these symptoms after treatment cessation (T4), suggesting these gains may be related to the natural course of symptom recovery. More importantly, EA was well tolerated with only mild AEs, with majority of the patients reporting favorable perceptions toward the intervention. Altogether, our data suggest that EA is a safe and potentially efficacious intervention for neuropsychiatric symptoms, and that targeted acupoint selection enables improving the management of cancer-related cognitive impairment (CRCI).

Our study was distinctive in its integration with both structural and functional imaging analyses, offering an additional layer of insight that enhances our understanding of the observed cognitive outcomes. Structural imaging analyses suggested that nEA mitigated chemotherapy-related gray matter volume loss compared with sEA, and correlation analyses within the nEA group revealed several statistically significant associations between cognitive outcomes and neuroimaging metrics, aligning with studies linking chemotherapy to structural brain changes and cognitive decline.^54,55^ Hippocampal T1-weighted intensity increases in the nEA group may reflect neuroplasticity or reduced inflammation, and protection against the progressive neurodegeneration consistent with preclinical evidence of acupuncture’s effects on hippocampal function and neurotrophic signaling.^56,57^ Within the nEA group, attention and QoL also correlated positively with brain structure, suggesting potential cognitive benefits. However, the findings are exploratory and require validation in larger, longitudinal studies to confirm nEA’s role in preserving brain structure and function during cancer treatment.

Our fMRI data provided additional context for the cognitive improvement we observed in this trial. Reduced connectivity between the DMN and SFG was associated with greater memory improvement in the nEA group, suggesting that decoupling from the “task-negative” DMN reduces interference from internally oriented processes and facilitates better engagement of task-relevant systems. In addition, increased connectivity within taskpositive control and sensory networks, including the DAN-S1 and right FPN-IFG, was associated with better memory performance in nEA, whereas the sEA group again exhibited the reverse pattern. These findings indicate that stronger communication between attentional, sensory, and executive regions supports effective cognitive processing when modulated adaptively, as in nEA, but may reflect inefficient recruitment when elevated inappropriately, as in sEA. Similarly, for response speed, in the nEA group, increased connectivity between the DAN-SMG and rFPN-SMG/S1 regions was associated with faster responses, suggesting enhanced integration of attentional control and sensorimotor processing that facilitates efficient stimulus-response mapping. The sEA group, however, showed the opposite association, consistent with less adaptive network modulation and potentially less efficient cognitive-motor integration.

Building upon the imaging findings from this study, the observed improvements in cognitive function among participants receiving the nEA regimen are similarly consistent even after adjusting for participants’ perceptions of their assigned treatment. Furthermore, the pre-to-post intervention changes from baseline were statistically significant at both T3 and T4 time points, even after correcting for multiple comparisons. These cognitive gains are further supported by neuroimaging biomarkers, which revealed a significant increase in gray matter volume following nEA treatment—an increase that demonstrated a moderate correlation with improvements in the attention domain. Our findings point to EA with targeted acupoint selection, based on a combination of scientific, clinical and acupuncturist expert knowledge, as a promising treatment for CRCI in breast cancer survivors. Specifically, these points include GV20 *Baihui*, EX-NH1 *Sishencong*, and ST36 *Zusanli*, with or without PC6 *Neiguan*, which are consistent with findings from randomized controlled acupuncture trials and preclinical studies.^19,58,59^

More than 80% of participants reported clinically significant improvement in at least one neuropsychiatric symptom after adjusting for treatment guesses. Furthermore, across PRO measures, the pre-post effect sizes (Glass’s Δ) for self-perceived cognition, fatigue, and QoL exceeded the standardized threshold of 0.5, indicating a clinically meaningful change relative to baseline variability in both groups.^60^ These effects may stem from shared components of the interventions, including a 30-min resting period in a supine position and electrical stimulation alongside needle insertion. The treatment allows participants to take a short daytime nap that promotes rejuvenation and enhances emotional well-being.^61^ The use of electrical stimulation in EA sends stronger stimuli that activate thinly myelinated and unmyelinated autonomic fibers known for high internal resistance.^62,63^ Thus, both nEA and sEA could have modulated the autonomic nervous system via the somato-autonomic reflex, increasing parasympathetic tone while reducing sympathetic activity, thereby promoting physiological balance and relaxation.^64,65^ Nevertheless, the value of acupoint selection remains evident from a significant improvement in distress symptoms with nEA treatment but not sEA, suggesting that stimulating nEA acupoints such as ST36 *Zusanli* is more effective at autonomic modulation, corroborating with preclinical findings.^12^

Trends in plasma biomarkers were highly variable, necessitating a larger sample size for more definitive conclusions. Descriptively, the nEA and sEA appeared to activate different somato-autonomic reflexes. The nEA treatment reduced pro-inflammatory IL-8 with minimal changes in BDNF, with a correlation between markers like IL-6 and cognitive improvement. This anti-inflammatory profile, coupled with a descriptive increase in IL-33, suggests activation of the vagal-adrenal reflex, whereby nEA stimulates the vagus nerve to trigger the hypothalamus-pituitary-adrenal axis and release adrenal catecholamines, reducing systemic inflammation.^12-14^ The upregulation of IL-33 post-nEA is relevant for brain health as it maintains gray matter integrity by promoting DNA repair and autophagic clearance.^66-69^ Conversely, sEA increased both TNF-α and BDNF, indicating the activation of the spinal sympathetic reflex that increases inflammatory activity during acute inflammation.^14^ BDNF regulation could be linked to sEA-induced inflammatory changes.^70,71^ Past studies have linked an upregulation in neuronal BDNF with higher TNF-α levels, possibly as a negative feedback mechanism in response to inflammation.^72-74^ Further, the concurrent BDNF upregulation with TNF-α suggests that this sEA-induced sympathetic reflex did not trigger significant pathological inflammation.^75^ Further studies must explore the complex relationship between BDNF, inflammation, and neuronal health after EA treatment.

We acknowledge several limitations in the study. Placebo acupuncture devices (eg, Streitberger needle) were not used to blind both acupuncturists and patients by simulating needle insertion while maintaining clinical inertness.^76^ We accepted these limitations to address a key critique that the selection of acupoints alone does not account for the effects of acupuncture, and that its efficacy is largely driven by placebo responses. By employing a noninert sham control (EA at nondisease-related acupoints), separating acupuncturist and assessor roles, adjusting for placebo effects based on patients’ guesses of treatment allocation, as well as using objective outcome measures, we have successfully piloted a novel explanatory trial framework aimed at generating high-quality evidence for acupuncture efficacy. Despite minor baseline differences (age at cancer diagnosis and highest education level) due to the small sample size, sensitivity analyses confirmed the durability of our primary findings. While we did not require a minimum symptom severity for study eligibility, this approach addresses the real-world complexity of symptom clustering and has facilitated the inclusion of symptomatic breast cancer survivors with mild impairment across multiple symptom domains who would otherwise be excluded by traditional moderate-to-severe cutoffs. This strategy is further validated by our baseline data, which shows our participants presented with more severe symptoms than the average breast cancer population.^33,77-79^

Our findings support further validation of EA’s role in treating CRCI and related neuropsychiatric symptoms in larger, multicentered phase III definitive trials. Specifically, a 3-arm randomized design comparing EA, traditional acupuncture, and a waitlist control is necessary to isolate the therapeutic contribution of electrical stimulation while accounting for natural symptom progression. Future research should also incorporate cost-effectiveness analyses and evaluate 1-year durability to provide the economic evidence required to justify expanding Medicare reimbursement beyond chronic lower back pain. Finally, by validating this standardized protocol across multiple centers, we can move toward broader clinical implementation and ensure accessible, evidencebased relief for breast cancer survivors experiencing cognitive impairment and neuropsychiatric symptom burden.

## Conclusion

In this robust pilot trial, 10 weeks of electroacupuncture targeting neuropsychiatric-related acupoints, compared with nondisease acupoints, improves neuropsychiatric symptoms in breast cancer survivors, supported by clinically relevant brain changes and reduced pro-inflammatory cytokines in plasma. Our study calls for the continual refinement of acupoint regimens based on clinical and mechanistic data. Developing standardized, symptom-specific acupoint protocols will facilitate consistency and reproducibility across practices. Findings also support the potential efficacy of EA for CRCI and related symptoms, warranting large, multicenter trials to validate these findings and explore underlying mechanisms.

## Supplementary Material

Supplementary Materials

Supplementary Methods

Supplementary material is available at *JNCI: Journal of the National Cancer Institute* online.

## Figures and Tables

**Table 1.** Characteristics of study participants at baseline.

Characteristics	Total (*n* = 35)	nEA (*n* ¼ 18)	sEA (*n* ¼ 17)	*P*
Age at recruitment, mean (SD)	58.2 (12.2)	60.8 (11.9)	55.4 (12.2)	.197
Age at cancer diagnosis, mean (SD)	50.0 (10.8)	55.8 (10.8)	48.1 (9.7)	.033*
Female, No. (%)	35 (100%)	18 (100%)	17 (100%)	—
Race/ethnicity, No. (%)				.371
Non-Hispanic White	23 (65.7%)	12 (66.7%)	11 (64.7%)	
Hispanic/Latino	1 (2.9%)	0 (0%)	1 (5.9%)	
Non-Hispanic Asian	6 (17.1%)	2 (11.1%)	4 (23.5%)	
Other	5 (14.3%)	4 (22.2%)	1 (5.9%)	
Highest education level, No. (%)				.038*
High school diploma or less	2 (5.7%)	2 (11.1%)	0 (0%)	
Attended college/associate’s degree/technical school	6 (17.1%)	5 (27.8%)	1 (5.9%)	
Bachelor’s degree	10 (28.6%)	2 (11.1%)	8 (47.1%)	
Master’s degree or more	17 (48.6%)	9 (50%)	8 (47.1%)	
Married, No. (%)	24 (68.6%)	14 (77.8%)	10 (58.8%)	.227
Employed, No. (%)	21 (60.0%)	10 (55.6%)	11 (64.7%)	.581
Stage of cancer, No. (%)	21 (60.0%)	12 (66.7%)	9 (52.9%)	.837
II	8 (22.9%)	4 (22.2%)	4 (23.5%)	
III	5 (14.3%)	2 (11.1%)	3 (17.7%)	
IV	1 (2.9%)	0 (0.0%)	1 (5.9%)	
Exposure to cancer systemic therapy and/or radiotherapy, No. (%)	33 (94.3%)	16 (88.9%)	17 (100%)	.486
Time from last treatment modality, No. (%)				1.000
Currently receiving	11 (31.4%)	6 (33.3%)	5 (29.4%)	
Within last 6 months	7 (20.0%)	4 (22.2%)	3 (17.6%)	
6-12 months ago	2 (5.7%)	1 (5.6%)	1 (5.9%)	
More than 12 months ago	15 (42.9%)	7 (38.9%)	8 (47.1%)	
Self-reported neuropsychiatric symptoms present at baseline, No. (%)				
Cognitive impairment	22 (62.9%)	11 (61.1%)	11 (64.7%)	.826
Fatigue	25 (71.4%)	12 (66.7%)	13 (76.5%)	.711
Insomnia	28 (80.0%)	15 (83.3%)	13 (76.5%)	.691
Psychological distress	24 (68.6%)	13 (72.2%)	11 (64.7%)	.632
Two or more symptoms present, No. (%)	30 (85.7%)	15 (83.3%)	15 (88.2%)	1.000
Baseline health outcomes, median (Q1, Q3)				
FACT-Cog total^a^	98 (70, 127)	105 (69, 123)	95 (74, 127)	.908
CANTAB attention^a^	0.90 (0.87, 0.93)	0.90 (0.87, 0.92)	0.91 (0.87, 0.94)	.668
CANTAB memory^b^	10 (5, 18)	11 (7, 23)	10 (4, 17)	.779
CANTAB response speed^b^	410 (367, 438)	382 (360, 451)	425 (391, 433)	.248
CANTAB executive function^b^	8 (4, 10)	9 (7, 11)	8 (4, 9)	.200
CANTAB multitask^b^	197 (90, 248)	203 (93, 320)	174 (90, 224)	.306
MFSI-SF total^b^	19 (7, 47)	22 (5, 53)	16 (10, 36)	.419
C30 emotional functioning (EF)^a^	67 (50, 92)	63 (42, 83)	83 (58, 92)	.148
C30 insomnia (SL)^b^	66.7 (33.3, 100)	66.7 (33.3, 91.7)	66.7 (33.3, 100)	.945
C30 global health status (GHS)^a^	67 (50, 75)	67 (50, 75)	58 (50, 67)	.485
Baseline plasma biomarkers,^c^ median (Q1, Q3)				
BDNF, pg/mL	1868 (952, 3407)	1285 (973, 3975)	2812 (623, 3407)	.740
TrkB, pg/mL	825 (534, 924)	762 (386, 1051)	827 (534, 903)	.683
CXCL13, pg/mL	138 (113, 193)	160 (113, 214)	132 (113, 150)	.254
IL-1α, pg/mL	9.5 (8.0, 10.8)	9.4 (8.4, 10.8)	9.5 (6.9, 10.9)	.604
IL-6, pg/mL	0 (0, 6)	0 (0, 5)	0 (0, 7)	.621
IL-8, pg/mL	6 (0, 11)	2 (0, 13)	6 (5, 11)	.460
IL-10, pg/mL	0.81 (0.56, 1.63)	1.01 (0.70, 1.63)	0.71 (0.23, 1.64)	.361
IL-17A, pg/mL	1.5 (0.0, 3.9)	2.5 (0.0, 3.7)	0.0 (0.0, 4.8)	.759
IL-18, pg/mL	226 (152, 314)	282 (166, 357)	197 (123, 300)	.126
IL-21, pg/mL	440 (362, 469)	435 (373, 470)	446 (289, 469)	.683
IL-33, pg/mL	0 (0, 0.038)	0 (0, 2.32)	0 (0, 0)	.523
MCP-1, pg/mL	183 (136, 213)	205 (136, 251)	177 (110, 209)	.213
MIP-1α, pg/mL	0 (0, 9)	0 (0, 8)	3 (0, 17)	.543
RANTES, pg/mL	1413 (890, 2148)	1488 (1156, 4279)	1404 (868, 1864)	.512
TNF-α, pg/mL	8 (3, 14)	7 (4, 14)	10 (0, 16)	.950

a Higher values represent better outcomes.

b Lower values represent better outcomes.

c IL-1β, IL-4, and IL-23 were excluded as >80% of values were below the lower limit of quantification.

* *P* <.05.

Abbreviations: BDNF = brain-derived neurotrophic factor; C30 = European Organisation for Research and Treatment of Cancer Core Quality of Life (quality of life GHS, psychological distress EF, insomnia SL); CANTAB = Cambridge Neuropsychological Test Automated Battery (objective cognition); CXCL13 = chemokine (C-X-C motif) ligand 13; FACT-Cog = Functional Assessment of Cancer Therapy—Cognitive Function version 3 (self-perceived cognition); IL = interleukin; MCP-1 = monocyte chemoattractant protein 1; MFSI-SF = Multidimensional Fatigue Syndrome Inventory-Short Form (fatigue); MIP-1α = macrophage inflammatory protein-1 alpha; nEA = neuropsychiatric-specific electroacupuncture; RANTES = regulated on activation, normal T-cell expressed and secreted; SD = standard deviation; sEA = sham electroacupuncture; TNF-α = tumor necrosis factor alpha; TrkB = tropomyosin receptor kinase B.

## Data Availability

The datasets generated and/or analyzed during the current study are not publicly deposited but are available from the corresponding author on reasonable request.
