# Supplementary material for: Electroacupuncture improves cognitive function and neuropsychiatric symptoms in breast cancer survivors: a pilot randomized controlled trial
Source: J Natl Cancer Inst. 2026 Apr 3;118(9):1692–704. doi: 10.1093/jnci/djag096 (PMC13285686; doi:10.1093/jnci/djag096)
Supplement: Supplementary file 1 [file djag096_supplementary_data.docx]

Supplementary Materials

***Electroacupuncture Improves Cognition and Distress Symptoms in Breast Cancer Survivors: A Pilot RCT***

**Figure S1: Spearman correlation matrix of pairwise outcome-biomarker comparisons, significant and non-significant.**

**Table S1: Electroacupuncture intervention for treating neuropsychiatric symptoms in breast cancer – scientific and clinical rationale.**

**Table S2: Selected CANTAB® cognitive measures.**

**Table S3: Health outcomes descriptive statistics.**

**Table S4: Changes in health outcomes from baseline to T3 and T4.**

**Table S5: Changes in health outcomes from baseline to T3 and T4, adjusted for treatment guesses.**

**Table S6: Changes in health outcomes from baseline to T3 and T4, adjusted for baseline differences in age at cancer diagnosis and highest education level.**

**Table S7: Distribution of treatment responders at T3 and T4.**

**Table S8: Plasma biomarkers descriptive statistics.**

**Table S9: Changes in plasma biomarkers from baseline to T3 and T4.**

**Table S10: Pearson’s correlation analysis of gray and white matter and hippocampal metrics with cognitive domains and quality of life at T3, stratified by treatment group.**

**Table S11: Associations between changes in brain connectivity and cognitive measures of memory and attention.**

**Table S12: Patient acceptance of EA and treatment blinding outcomes.**

**Figure S1: Spearman correlation matrix of pairwise outcome-biomarker comparisons.** Changes in health outcomes were standardized so that positive values correspond to an improved outcome from baseline.

**Table S1: Electroacupuncture intervention for treating neuropsychiatric symptoms in breast cancer – scientific and clinical rationale.**

| **Neuropsychiatric-specific EA (nEA) acupoints** | **Location** |
| --- | --- |
| Shenting (GV24) | On the head, 0.5 B-cun^1^ superior to the anterior hairline, on the anterior median line. When the anterior hairline is unclear or changed, GV 24 is located 3.5 B-cun superior to the midpoint between the medial ends of the eyebrows. |
| Baihui (GV20) | On the head, 5 B-cun superior to the anterior hairline on the anterior median line. |
| Sishencong (EX-HN1) | A group of four points at the vertex, 1 B-cun from GV20 in a cross-formation |
| Zhongwan (CV12) | On the upper abdomen, 4 B-cun superior to the center of the umbilicus, on the anterior median line. |
| Guanyuan (CV4) | On the lower abdomen, 3 B-cun inferior to the center of the umbilicus, on the anterior median line. |
| Neiguan (PC6) | On the anterior aspect of the forearm, between the tendons of the palmaris longus and the flexor carpi radialis, 2 B-cun proximal to the palmar wrist crease |
| Shenmen (HT7) | On the anteromedial aspect of the wrist, radial to the flexor carpi ulnaris tendon, on the palmar wrist crease. |
| Hegu (LI4) | On the dorsum of the hand, radial to the midpoint of the second metacarpal bone |
| Zusanli (ST36) | On the anterior aspect of the leg, about one finger-breadth lateral to the tibia, 3 B-cun inferior to ST35 (On the anterior aspect of the knee, in the depression lateral to the patellar ligament). |
| Sanyinjiao (SP6) | On the tibial aspect of the leg, posterior to the medial border of the tibia, 3 B-cun superior to the prominence of the medial malleolus. |
| Taixi (KI3) | On the posteromedial aspect of the ankle, in the depression between the prominence of the medial malleolus and the calcaneal tendon. |
| Zhaohai (KI6) | On the medial aspect of the foot, 1 B-cun inferior to the prominence of the medial malleolus, in the depression inferior to the medial malleolus. |
| Taichong (LIV3) | On the dorsum of the foot, between the first and second metatarsal bones, in the depression distal to the junction of the bases of the two bones, over the dorsalis pedis artery. |
| ^1^This method uses landmarks on the body surface, such as joints, divides the length between two points into equal portions and locates acupuncture points by such proportions. Each portion equals 1 B-cun. | |
| **Intervention design** | **Rationale** |
| Electrical stimulation with acupuncture | - To activate thinly myelinated and unmyelinated autonomic fibers known for high internal resistance for modulating the autonomic nervous system [1-4]. - The anti-inflammatory and neuromodulating effects of acupuncture are largely observed preclinically with electroacupuncture (EA) [5-7]. |
| Neuropsychiatric-specific EA (nEA) acupoints: *Shenting* (GV24), *Baihui* (GV20), *Sinshencong* (EX-HN1), *Zhongwan* (CV12), *Guanyuan* (CV4), *Neiguan* (PC6) bilateral, *Shenmen* (HT7) bilateral, *Zusanli* (ST36) bilateral, *Sanyinjiao* (SP6) bilateral, *Taixi* (KI3) bilateral, *Zhaohai* (KI6) bilateral, *Hegu* (LI4) bilateral, *Taichong* (LIV3) bilateral | Clinical (cancer-specific)   - In breast cancer patients with cancer-related cognitive impairment (CRCI), acupuncture [*Baihui* (GV20), *Sishencong* (EX-HN1), and *Taixi* (KI3)] improves memory and executive function through BDNF upregulation based on trial findings (n=80) from Tong et al, 2018 [8]. - In randomized trial comparing acupuncture against cognitive behavior therapy (CBT) for insomnia treatment among cancer survivors (n=160), acupuncture treatment at *Shenmen* (HT7), *Sanyinjiao* (SP6), *Baihui* (GV20), and *Shenting* (DU24) were found with comparable and clinically significant improvement in insomnia with CBT. Both groups also reported similar improvements in fatigue, mood, and quality of life and reduced prescription hypnotic medication use [9]. Cancer survivors who reported concurrent insomnia symptoms and CRCI (n=99) were found with comparable improvement in objective attention (acupuncture vs CBT), but a statistically significant pre-post enhancement in learning and memory function with acupuncture that is not observed with CBT [10]. Further analysis found a statistically significant increase in serum brain-derived neurotrophic factor (BDNF) levels after acupuncture treatment, but not with CBT among participants with low baseline levels [11]. Finally, patients with both insomnia and fatigue were also observed with clinically and statistically significant improvement after receiving either acupuncture or CBT [12]. Acupuncture and CBT have comparable efficacy in treating multiple neuropsychiatric symptoms experienced by cancer survivors. - In a feasibility pilot trial evaluating acupuncture against sham and waitlist controls among cancer survivors, treatment at *Baihui* (GV20), *Sishencong* (EX-HN1), *Shenmen* (HT7), *Sanyinjiao* (SP6), *Taixi* (KI3), and *Zhongwan* (CV12), significantly improves self-perceived cognition from, with the largest improvement observed among true acupuncture, followed by sham control and finally waitlist control [13]. |
|  | Science (rodent models)   - In rodent models, EA at hindlimb *Zusanli* (ST36) stimulates the vagus nerve and the adrenal gland to release catecholamines (e.g., norepinephrine and dopamine) to counteract systemic inflammation by reducing pro-inflammatory interleukin-6 (IL-6) and tumor necrosis factor alpha (TNF-α) [5-7]. - EA treatment in rodent models, at *Baihui* (GV20), *Shenmen* (HT7), and *Sanyinjiao* (SP6), have been shown to alleviate insomnia, distress, and cognitive impairment via the upregulation of brain-derived neurotrophic factor (BDNF) [14-17]. - EA at *Guanyuan* (CV4), *Zusanli* (ST36), and *Baihui* (GV20) modulates aging-related genes in the hippocampus [18]. |
|  | Science (neuroimaging)   - Acupuncture treatment on wrist *Neiguan* (PC6) increases functional connectivity between the hippocampus and the default mode network facilitated by enhanced parasympathetic tone and inhibited sympathetic nervous system [19]. - Acupuncture treatment of patients with amnestic mild cognitive impairment at bilateral acupoints Hegu (LI4) and Taichong (LIV3) increases functional connectivity of the hippocampus with middle and inferior temporal gyri of the brain that are responsible for cognitive processes such as language processing, visual object recognition, and associative learning [20]. |
|  | Clinical (non-cancer)   - Acupuncture with moxibustion treatment at *Zhaohai* (KI6) in combination with *Sishencong* (EX-HN1), *Neiguan* (PC6), *Shenmen* (HT7), *Zusanli* (ST36), *Sanyinjiao* (SP6), *Taichong* (LIV3) improved depressive symptoms in patients with post-stroke depression [21]. |
| Sham EA (sEA): *Pianli* (LI6) bilateral, *Wenliu* (LI7) bilateral, *Fuyang* (BL59) bilateral, *Kunlun* (BL60) bilateral, *Sanyangluo* (TE8), *Sidu* (TE9) bilateral, *Daheng* (SP15) bilateral | - None of these acupoints have shown effectiveness with neuropsychiatric symptom management. These were selected by acupuncturists based on their clinical expertise. |

References:

1. Glatte P, Buchmann SJ, Hijazi MM, Illigens BMW, Siepmann T. Architecture of the Cutaneous Autonomic Nervous System. Front Neurol. 2019;10. doi:10.3389/FNEUR.2019.00970
2. Andersson S, Lundeberg T. Acupuncture--from empiricism to science: functional background to acupuncture effects in pain and disease. Med Hypotheses. 1995;45(3):271-281. doi:10.1016/0306-9877(95)90117-5
3. Uchida S, Budgell B. Somato-Autonomic Reflex. Encyclopedia of Neuroscience. Published online 2009:3767-3770. doi:10.1007/978-3-540-29678-2_5500
4. Sato A. Neural mechanisms of autonomic responses elicited by somatic sensory stimulation. Neurosci Behav Physiol. 1997;27(5):610-621. doi:10.1007/BF02463910
5. Liu S, Wang Z, Su Y, et al. A neuroanatomical basis for electroacupuncture to drive the vagal–adrenal axis. Nature 2021 598:7882. 2021;598(7882):641-645. doi:10.1038/s41586-021-04001-4
6. Torres-Rosas R, Yehia G, Peña G, et al. Dopamine mediates vagal modulation of the immune system by electroacupuncture. Nature Medicine 2014 20:3. 2014;20(3):291-295. doi:10.1038/nm.3479
7. Liu S, Wang ZF, Su YS, et al. Somatotopic Organization and Intensity Dependence in Driving Distinct NPY-Expressing Sympathetic Pathways by Electroacupuncture. Neuron. 2020;108(3):436-450.e7. doi:10.1016/J.NEURON.2020.07.015/ATTACHMENT/1FA9A77C-29BE-4D00-B7F5-89EC0C1B23C8/MMC2.PDF
8. Tong T, Pei C, Chen J, Lv Q, Zhang F, Cheng Z. Efficacy of acupuncture therapy for chemotherapy-related cognitive impairment in breast cancer patients. Medical Science Monitor. 2018;24:2919-2927. doi:10.12659/MSM.909712
9. Garland SN, Xie SX, DuHamel K, Bao T, Li Q, Barg FK, Song S, Kantoff P, Gehrman P, Mao JJ. Acupuncture Versus Cognitive Behavioral Therapy for Insomnia in Cancer Survivors: A Randomized Clinical Trial. J Natl Cancer Inst. 2019 Dec 1;111(12):1323-1331. doi: 10.1093/jnci/djz050.
10. Liou KT, Root JC, Garland SN, et al. Effects of acupuncture versus cognitive behavioral therapy on cognitive function in cancer survivors with insomnia: A secondary analysis of a randomized clinical trial. Cancer. 2020;126(13):3042-3052. doi:10.1002/CNCR.32847
11. Liou KT, Garland SN, Li QS, et al. Effects of acupuncture versus cognitive behavioral therapy on brain-derived neurotrophic factor in cancer survivors with insomnia: an exploratory analysis. Acupuncture in medicine : journal of the British Medical Acupuncture Society. 2021;39(6):637-645. doi:10.1177/0964528421999395
12. Li X, Liou KT, Chimonas S, et al. Addressing cancer-related fatigue through sleep: A secondary analysis of a randomized trial comparing acupuncture and cognitive behavioral therapy for insomnia. Integr Med Res. 2023;12(1). doi:10.1016/J.IMR.2023.100922
13. Li X, Lampson K, Ahles TA, Root JC, Li QS, Li Y, Ahsan A, Mao JJ, Liou KT. Feasibility and Preliminary Effects of Acupuncture for Cognitive Dysfunction in Diverse Cancer Survivors: A Pilot, Randomized, Placebo-Controlled Trial. Curr Oncol. 2025 Jan 1;32(1):27. doi: 10.3390/curroncol32010027.
14. Qiao L, Shi Y, Tan L, Jiang Y, Yang Y. Efficacy of electroacupuncture stimulating Shenmen (HT7), Baihui (GV20), Sanyinjiao (SP6) on spatial learning and memory deficits in rats with insomnia induced by para-chlorophenylalanine: a single acupoint combined acupoints. J Tradit Chin Med. 2023;43(4):704-714. doi:10.19852/J.CNKI.JTCM.20230308.001
15. Cao F, Xu Y, Zhang M, et al. Baihui (DU20), Shenmen (HT7) and Sanyinjiao (SP6) target the cAMP/CREB/BDNF and PI3K/Akt pathways to reduce central nervous system apoptosis in rats with insomnia. Heliyon. 2022;8(12). doi:10.1016/J.HELIYON.2022.E12574
16. Lin R, Li L, Zhang Y, et al. Electroacupuncture ameliorate learning and memory by improving N-acetylaspartate and glutamate metabolism in APP/PS1 mice. Biol Res. 2018;51(1). doi:10.1186/S40659-018-0166-7
17. Duan DM, Tu Y, Liu P, Jiao S. Antidepressant effect of electroacupuncture regulates signal targeting in the brain and increases brain-derived neurotrophic factor levels. Neural Regen Res. 2016;11(10):1595-1602. doi:10.4103/1673-5374.193238
18. Liu J, Liu J, Wang G, Liu G, Zhou H, Fan Y, Liang F, Wang H. Electroacupuncture at Guanyuan (CV 4), Zusanli (ST 36) and Baihui (DU 20) regulate the aging-related changes in gene expression profile of the hippocampus in sub-acutely aging rats. PLoS One. 2018 Jan 19;13(1):e0191623. doi: 10.1371/journal.pone.0191623.
19. Dhond RP, Yeh C, Park K, Kettner N, Napadow V. Acupuncture modulates resting state connectivity in default and sensorimotor brain networks. *Pain*. 2008;136(3):407-418. doi:10.1016/J.PAIN.2008.01.011
20. Li H, Wang Z, Yu H, et al. The Long-Term Effects of Acupuncture on Hippocampal Functional Connectivity in aMCI with Hippocampal Atrophy: A Randomized Longitudinal fMRI Study. Neural Plast. 2020;2020. doi:10.1155/2020/6389368
21. Liu SK, Zhao XM, Xi ZM. [Incidence rate and acupuncture-moxibustion treatment of post-stroke depression]. Zhongguo Zhen Jiu. 2006 Jul;26(7):472-4. Chinese.

**Table S2: Selected CANTAB® cognitive measures.**

| **Cognitive domains** | **Key measure description** |
| --- | --- |
| Multitasking (MTT) | Multitasking cost (median) (MTTMTCMD): The difference between the median latency of response (from stimulus appearance to button press) during assessed blocks in which both rules are used versus assessed blocks in which only a single rule is used. Calculated by subtracting the median latency of response during single task block(s) from the median latency of response during multitasking block(s). A positive score indicates that the subject responds more slowly during multitasking blocks, and indicates a higher cost of managing multiple sources of information. |
| Memory (PAL) | PAL Total Errors (Adjusted) (PALTEA28): The number of times the subject chose the incorrect box for a stimulus on assessment problems (PALTE), plus an adjustment for the estimated number of errors they would have made on any problems, attempts and recalls they did not reach. This measure allows you to compare performance on errors made across all subjects regardless of those who terminated early versus those completing the final stage of the task. In this task variant PALTEA does not include 12 box level to provide a direct comparison to Recommended Standard. |
| Response speed (RTI) | RTI Median Five-Choice Reaction Time (RTIFMDRT): The median duration it took for a subject to release the response button after the presentation of a target stimulus. Calculated across correct, assessed trials in which the stimulus could appear in any one of five locations. Measured in milliseconds. |
| Executive function (SWM) | SWM Strategy (6-8 boxes) (SWMS): The number of times a subject begins a new search pattern from the same box they started with previously. If they always begin a search from the same starting point we infer that the subject is employing a planned strategy for finding the tokens. Therefore a low score indicates high strategy use (1 = they always begin the search from the same box), a high score indicates that they are beginning their searches from many different boxes. Calculated across assessed trials with 6 tokens or 8 tokens. |
| Attention (RVP) | RVP A prime (A’) (RVP A’): A' is the signal detection measure of sensitivity to the target, regardless of response tendency (expected range 0.000 to 1.000; bad to good). In essence, this metric is a measure of how good the subject is at detecting target sequences. |

**Table S3: Health outcomes descriptive statistics.**

|  | **EA Treatment** | **T1** | **T2** | **T3** | **T4** |
| --- | --- | --- | --- | --- | --- |
| Outcome measures, mean (SD) |  |  |  |  |  |
| FACT-Cog total^a^ | sEA | 92.9 (39.4) | 114 (31.3) | 119 (24) | 119 (26.7) |
|  | nEA | 96 (36.2) | 112 (28.3) | 122 (19.2) | 126 (18.8) |
| Attention raw^a^ | sEA | 0.895 (0.062) | 0.924 (0.049) | 0.921 (0.074) | 0.933 (0.073) |
|  | nEA | 0.892 (0.044) | 0.920 (0.057) | 0.933 (0.044) | 0.920 (0.049) |
| Attention RCI^a^ | sEA | - | 0 (1) | -0.1 (2.02) | 0.3 (2.05) |
|  | nEA | - | -0.13 (1.39) | 0.32 (0.9) | 0.66 (0.67) |
| Memory raw^b^ | sEA | 13.3 (12.0) | 9.31 (10.6) | 10.3 (12.1) | 10.0 (13.0) |
|  | nEA | 16.4 (16.4) | 10.5 (13.3) | 8.93 (8.54) | 11.1 (10.9) |
| Memory RCI^a^ | sEA | - | 0 (1) | -0.11 (1.06) | -0.08 (1.1) |
|  | nEA | - | -0.08 (0.94) | 0.14 (0.5) | -0.09 (0.98) |
| Response speed raw^b^ | sEA | 415 (36.3) | 406 (43.8) | 412 (63.9) | 404 (52.7) |
|  | nEA | 407 (71.8) | 376 (41.5) | 376 (32.9) | 379 (42.1) |
| Response speed RCI^a^ | sEA | - | 0 (1) | -0.16 (1.54) | 0.05 (1.29) |
|  | nEA | - | 0.5 (1.23) | 0.51 (1.13) | 0.42 (0.9) |
| Executive function raw^b^ | sEA | 7.12 (3.31) | 7.38 (3.28) | 7.06 (3.51) | 6.69 (3.63) |
|  | nEA | 8.22 (3.19) | 6.93 (3.33) | 7.36 (4.05) | 7.50 (3.61) |
| Executive function RCI^a^ | sEA | - | 0 (1) | 0.15 (0.92) | 0.34 (1.22) |
|  | nEA | - | 0.36 (0.79) | 0.26 (1.46) | 0.19 (1.36) |
| Multitasking raw^b^ | sEA | 162 (111) | 178 (102) | 153 (64.4) | 130 (100) |
|  | nEA | 203 (119) | 148 (123) | 145 (121) | 116 (117) |
| Multitasking RCI^a^ | sEA | - | 0 (1) | 0.24 (0.63) | 0.41 (0.97) |
|  | nEA | - | 0.29 (1.18) | 0.32 (1.17) | 0.61 (1.13) |
| MFSI-SF total^b^ | sEA | 22.5 (26.6) | 1.3 (16.1) | -0.1 (15.2) | -2.5 (18.6) |
|  | nEA | 30.1 (28.1) | 16.7 (27.6) | 3.4 (28.6) | 3.6 (18.7) |
| EORTC QLQ-C30 GHS^a^ | sEA | 60.3 (21.6) | 73.4 (18.8) | 75 (16.9) | 77.6 (19.7) |
|  | nEA | 61.6 (27.4) | 67.2 (23.2) | 80.4 (12.5) | 75.6 (10.6) |
| EORTC QLQ-C30 EF^a^ | sEA | 72.5 (22.8) | 81.3 (21.8) | 83.9 (25.2) | 86.5 (19.7) |
|  | nEA | 58.8 (28.5) | 68.9 (25.1) | 82.7 (13.7) | 81.5 (18.0) |
| EORTC QLQ-C30 SL^b^ | sEA | 56.9 (34.9) | 43.7 (35.9) | 39.6 (32.7) | 27.1 (27.8) |
|  | nEA | 57.4 (35.8) | 51.1 (27.8) | 42.9 (33.1) | 50.0 (28.5) |
| Abbreviations: CANTAB, Cambridge Neuropsychological Test Automated Battery (objective cognition); EORTC QLQ-C30, European Organization for Research and Treatment of Cancer Core Quality of Life (quality of life GHS, psychological distress EF, insomnia SL); FACT-Cog, Functional Assessment of Cancer Therapy—Cognitive Function version 3 (self-perceived cognition); MFSI-SF, Multidimensional Fatigue Syndrome Inventory-Short Form (fatigue); nEA, neuropsychiatric-specific electroacupuncture; SD, standard deviation; sEA, sham electroacupuncture.  ^a^ Higher values represent better outcomes.  ^b^ Lower values represent better outcomes. | | | | | |

**Table S4: Changes in health outcomes from baseline to T3 and T4.**

| **Symptom domains** | **Outcome measures (timepoints)** | **Baseline-adjusted mean change** | | **Between-group differences** |
| --- | --- | --- | --- | --- |
|  |  | **Effect size^a^ (95% CI)** | | **Cohen’s *d*^b^** |
|  |  | **nEA** | **sEA** | **nEA vs sEA** |
| Self-perceived cognition | FACT-Cog total |  |  |  |
|  | T3 – T1 | ***0.685* (0.338, 1.031)*** | ***0.730* (0.395, 1.064)*** | -0.051 |
|  | T4 – T1 | ***0.823* (0.476, 1.169)*** | ***0.728* (0.393, 1.062)*** | 0.333 |
| Objective cognition | CANTAB attention |  |  |  |
|  | T3 – T1 | ***0.562* (0.147, 0.977)*** | 0.368 (-0.031, 0.767) | 0.425 |
|  | T4 – T1 | ***0.708* (0.293, 1.123)*** | ***0.542* (0.143, 0.941)*** | 0.360 |
|  | CANTAB memory |  |  |  |
|  | T3 – T1 | 0.544 (0.033, 1.055) | 0.256 (-0.239, 0.751) | 0.400 |
|  | T4 – T1 | 0.377 (-0.135, 0.888) | 0.281 (-0.214, 0.775) | 0.045 |
|  | CANTAB response speed |  |  |  |
|  | T3 – T1 | 0.341 (-0.072, 0.729) | 0.038 (-0.357, 0.434) | 0.805 |
|  | T4 – T1 | 0.294 (-0.118, 0.706) | 0.150 (-0.246, 0.545) | 0.438 |
|  | CANTAB executive function |  |  |  |
|  | T3 – T1 | 0.224 (-0.240, 0.687) | 0.108 (-0.338, 0.554) | 0.165 |
|  | T4 – T1 | 0.180 (-0.283, 0.644) | 0.223 (-0.224, 0.669) | -0.161 |
|  | CANTAB multitask |  |  |  |
|  | T3 – T1 | 0.309 (-0.337, 0.955) | 0.063 (-0.559, 0.686) | 0.120 |
|  | T4 – T1 | 0.563 (-0.083, 1.209) | 0.215 (-0.407, 0.837) | 0.271 |
| Fatigue | MFSI-SF total |  |  |  |
|  | T3 – T1 | ***0.978* (0.588, 1.368)*** | ***0.853* (0.477, 1.229)*** | 0.110 |
|  | T4 – T1 | ***0.969* (0.579, 1.359)*** | ***0.956* (0.580, 1.332)*** | -0.164 |
| Psychological distress | C30 emotional functioning (EF) |  |  |  |
|  | T3 – T1 | ***0.764* (0.239, 1.289)*** | 0.390 (-0.112, 0.893) | 0.379 |
|  | T4 – T1 | ***0.711* (0.186, 1.236)*** | 0.506 (0.004, 1.009) | 0.070 |
| Insomnia | C30 insomnia (SL) |  |  |  |
|  | T3 – T1 | 0.371 (-0.303, 1.044) | 0.459 (-0.192, 1.111) | -0.135 |
|  | T4 – T1 | 0.160 (-0.514, 0.834) | ***0.829* (0.177, 1.480)*** | ***-0.950^†^*** |
| Quality of life | C30 global health status (GHS) |  |  |  |
|  | T3 – T1 | ***0.681* (0.221, 1.141)*** | ***0.568* (0.122, 1.013)*** | 0.259 |
|  | T4 – T1 | 0.464 (0.004, 0.924) | ***0.686* (0.241, 1.132)*** | -0.430 |
| Abbreviations: CANTAB, Cambridge Neuropsychological Test Automated Battery; CI, confidence interval; EORTC QLQ-C30, European Organisation for Research and Treatment of Cancer Core Quality of Life; FACT-Cog, Functional Assessment of Cancer Therapy—Cognitive Function version 3; nEA, neuropsychiatric-specific electroacupuncture; sEA, sham electroacupuncture.  ^a^ Glass’s Δ. Greater than 0 indicates improvement from baseline while less than 0 indicates worsening from baseline. Values of 0.2, 0.5, and 0.8 reflect small, medium, and large effect sizes, respectively.  ^b^ Cohen’s *d*. Greater than 0 indicates a larger improvement for nEA while less than 0 indicates a smaller improvement for nEA, relative to sEA. Values of 0.2, 0.5, and 0.8 reflect small, medium, and large effect sizes, respectively.  * p-adjusted < 0.05, controlling for multiple testing using the Benjamini-Hochberg method.  † p < 0.05 for Cohen’s d. | | | | |

**Table S5: Changes in health outcomes from baseline to T3 and T4, adjusted for treatment guesses.**

| **Symptom domains** | **Outcome measures (timepoints)** | **Baseline-adjusted mean change** | | **Between-group differences** |
| --- | --- | --- | --- | --- |
|  |  | **Effect size^a^ (95% CI)** | | **Cohen’s *d*^b^** |
|  |  | **nEA** | **sEA** | **nEA vs sEA** |
| Self-perceived cognition | FACT-Cog total |  |  |  |
|  | T3 – T1 | ***0.648* (0.284, 1.012)*** | ***0.700* (0.360, 1.041)*** | 0.031 |
|  | T4 – T1 | ***0.786* (0.422, 1.150)*** | ***0.698* (0.358, 1.039)*** | 0.414 |
| Objective cognition | CANTAB attention |  |  |  |
|  | T3 – T1 | ***0.553* (0.114, 0.991)*** | 0.366 (-0.044, 0.776) | 0.431 |
|  | T4 – T1 | ***0.698* (0.260, 1.137)*** | ***0.540* (0.130, 0.950)*** | 0.367 |
|  | CANTAB memory |  |  |  |
|  | T3 – T1 | 0.535 (-0.007, 1.077) | 0.263 (-0.244, 0.770) | 0.363 |
|  | T4 – T1 | 0.368 (-0.174, 0.910) | 0.288 (-0.219, 0.794) | 0.013 |
|  | CANTAB response speed |  |  |  |
|  | T3 – T1 | 0.306 (-0.125, 0.737) | 0.035 (-0.368, 0.438) | 0.854 |
|  | T4 – T1 | 0.260 (-0.171, 0.691) | 0.147 (-0.257, 0.550) | 0.491 |
|  | CANTAB executive function |  |  |  |
|  | T3 – T1 | 0.218 (-0.272, 0.709) | 0.115 (-0.344, 0.573) | 0.155 |
|  | T4 – T1 | 0.175 (-0.316, 0.665) | 0.229 (-0.229, 0.688) | -0.165 |
|  | CANTAB multitask |  |  |  |
|  | T3 – T1 | 0.210 (-0.472, 0.891) | 0.058 (-0.579, 0.696) | 0.280 |
|  | T4 – T1 | 0.464 (-0.217, 1.146) | 0.210 (-0.427, 0.848) | 0.429 |
| Fatigue | MFSI-SF total |  |  |  |
|  | T3 – T1 | ***0.961* (0.553, 1.368)*** | ***0.830* (0.449, 1.211)*** | -0.096 |
|  | T4 – T1 | ***0.952* (0.544, 1.359)*** | ***0.933* (0.552, 1.314)*** | 0.176 |
| Psychological distress | C30 emotional functioning (EF) |  |  |  |
|  | T3 – T1 | ***0.768* (0.248, 1.288)*** | 0.371 (-0.116, 0.857) | 0.383 |
|  | T4 – T1 | ***0.715* (0.195, 1.235)*** | 0.487 (0.000, 0.973) | 0.062 |
| Insomnia | C30 insomnia (SL) |  |  |  |
|  | T3 – T1 | 0.352 (-0.361, 1.064) | 0.431 (-0.236, 1.097) | 0.203 |
|  | T4 – T1 | 0.141 (-0.572, 0.854) | ***0.800* (0.133, 1.467)*** | ***-1.009^†^*** |
| Quality of life | C30 global health status (GHS) |  |  |  |
|  | T3 – T1 | ***0.650* (0.165, 1.135)*** | ***0.545* (0.091, 0.999)*** | 0.327 |
|  | T4 – T1 | 0.433 (-0.052, 0.918) | ***0.664* (0.210, 1.117)*** | -0.357 |
| Abbreviations: CANTAB, Cambridge Neuropsychological Test Automated Battery; CI, confidence interval; EORTC QLQ-C30, European Organisation for Research and Treatment of Cancer Core Quality of Life; FACT-Cog, Functional Assessment of Cancer Therapy—Cognitive Function version 3; N, counts; nEA, neuropsychiatric-specific electroacupuncture; sEA, sham electroacupuncture.  ^a^ Glass’s Δ. Greater than 0 indicates improvement from baseline while less than 0 indicates worsening from baseline. Values of 0.2, 0.5, and 0.8 reflect small, medium, and large effect sizes, respectively.  ^b^ Cohen’s *d*. Greater than 0 indicates a larger improvement for nEA while less than 0 indicates a smaller improvement for nEA, relative to sEA. Values of 0.2, 0.5, and 0.8 reflect small, medium, and large effect sizes, respectively.  * p-adjusted < 0.05, controlling for multiple testing using the Benjamini-Hochberg method.  † p < 0.05 for Cohen’s d. | | | | |

**Table S6: Changes in health outcomes from baseline to T3 and T4, adjusted for baseline differences in age at cancer diagnosis and highest education level.**

| **Symptom domains** | **Outcome measures (timepoints)** | **Baseline-adjusted mean change** | | **Between-group differences** |
| --- | --- | --- | --- | --- |
|  |  | **Effect size^a^ (95% CI)** | | **Cohen’s *d*^b^** |
|  |  | **nEA** | **sEA** | **nEA vs sEA** |
| Self-perceived cognition | FACT-Cog total |  |  |  |
|  | T3 – T1 | ***0.674 (0.326, 1.022)**** | ***0.728 (0.394, 1.063)**** | -0.199 |
|  | T4 – T1 | ***0.812 (0.464, 1.160)**** | ***0.726 (0.392, 1.061)**** | 0.186 |
| Objective cognition | CANTAB attention |  |  |  |
|  | T3 – T1 | ***0.554 (0.136, 0.972)**** | 0.372 (-0.028, 0.773) | 0.472 |
|  | T4 – T1 | ***0.700 (0.282, 1.118)**** | ***0.546 (0.146, 0.946)**** | 0.407 |
|  | CANTAB memory |  |  |  |
|  | T3 – T1 | 0.515 (0.001, 1.030) | 0.255 (-0.239, 0.750) | 0.524 |
|  | T4 – T1 | 0.348 (-0.166, 0.863) | 0.280 (-0.215, 0.775) | 0.170 |
|  | CANTAB response speed |  |  |  |
|  | T3 – T1 | 0.340 (-0.072, 0.753) | 0.040 (-0.355, 0.435) | ***1.179^†^*** |
|  | T4 – T1 | 0.294 (-0.118, 0.706) | 0.152 (-0.243, 0.546) | 0.812 |
|  | CANTAB executive function |  |  |  |
|  | T3 – T1 | 0.222 (-0.245, 0.689) | 0.104 (-0.363, 0.551) | 0.105 |
|  | T4 – T1 | 0.178 (-0.289, 0.645) | 0.218 (-0.229, 0.666) | -0.220 |
|  | CANTAB multitask |  |  |  |
|  | T3 – T1 | 0.312 (-0.339, 0.962) | 0.067 (-0.556, 0.691) | 0.355 |
|  | T4 – T1 | 0.566 (-0.084, 1.216) | 0.219 (-0.405, 0.843) | 0.506 |
| Fatigue | MFSI-SF total |  |  |  |
|  | T3 – T1 | ***0.960 (0.568, 1.352)**** | ***0.855 (0.479, 1.231)**** | -0.118 |
|  | T4 – T1 | ***0.951 (0.559, 1.343)**** | ***0.958 (0.582, 1.333)**** | -0.392 |
| Psychological distress | C30 emotional functioning (EF) |  |  |  |
|  | T3 – T1 | ***0.716 (0.193, 1.238)**** | 0.404 (-0.096, 0.903) | 0.459 |
|  | T4 – T1 | ***0.663 (0.140, 1.185)**** | 0.519 (0.020, 1.019) | 0.148 |
| Insomnia | C30 insomnia (SL) |  |  |  |
|  | T3 – T1 | 0.337 (-0.339, 1.013) | 0.456 (-0.195, 1.106) | -0.308 |
|  | T4 – T1 | 0.126 (-0.550, 0.802) | ***0.825 (0.175, 1.475)**** | ***-1.125^†^*** |
| Quality of life | C30 global health status (GHS) |  |  |  |
|  | T3 – T1 | ***0.632 (0.171, 1.092)**** | ***0.579 (0.134, 1.023)**** | 0.353 |
|  | T4 – T1 | 0.415 (-0.045, 0.875) | ***0.697 (0.253, 1.142)**** | -0.337 |
| Abbreviations: CANTAB, Cambridge Neuropsychological Test Automated Battery; CI, confidence interval; EORTC QLQ-C30, European Organisation for Research and Treatment of Cancer Core Quality of Life; FACT-Cog, Functional Assessment of Cancer Therapy—Cognitive Function version 3; N, counts; nEA, neuropsychiatric-specific electroacupuncture; sEA, sham electroacupuncture.  ^a^ Glass’s Δ. Greater than 0 indicates improvement from baseline while less than 0 indicates worsening from baseline. Values of 0.2, 0.5, and 0.8 reflect small, medium, and large effect sizes, respectively.  ^b^ Cohen’s *d*. Greater than 0 indicates a larger improvement for nEA while less than 0 indicates a smaller improvement for nEA, relative to sEA. Values of 0.2, 0.5, and 0.8 reflect small, medium, and large effect sizes, respectively.  * p-adjusted < 0.05, controlling for multiple testing using the Benjamini-Hochberg method.  † p < 0.05 for Cohen’s d. | | | | |

**Table S7: Distribution of treatment responders at T3 and T4.**

|  | **After treatment completion, T3** | | | **~4 weeks after treatment completion, T4** | | |
| --- | --- | --- | --- | --- | --- | --- |
|  | **nEA (N=14)** | **sEA (N=16)** | ***p*** | **nEA (N=14)** | **sEA (N=16)** | ***P*** |
| Treatment Responders^a^, n (%) |  |  |  |  |  |  |
| Objective cognitive function | 6 (42.9%) | 2 (12.5%) | 0.101 | 5 (35.7%) | 2 (12.5%) | 0.204 |
| Subjective cognitive function | 6 (42.9%) | 9 (56.3%) | 0.715 | 9 (64.3%) | 10 (62.5%) | 1.000 |
| Fatigue | 10 (71.4%) | 11 (68.8%) | 1.000 | 10 (71.4%) | 13 (81.3%) | 0.675 |
| Psychological distress | 7 (50%) | 6 (37.5%) | 0.713 | 7 (50%) | 5 (31.3%) | 0.457 |
| Insomnia | 7 (50%) | 7 (43.8%) | 1.000 | 6 (42.9%) | 8 (50%) | 0.730 |
| Improved in ≥1 symptom(s) | 13 (92.9%) | 13 (81.3%) | 0.602 | 12 (85.7%) | 15 (93.8%) | 0.586 |
| Participants with clinically meaningful improvement in quality of life^a^, n (%) | 8 (57.1%) | 7 (43.8%) | 0.715 | 4 (28.6%) | 7 (43.8%) | 0.466 |
| Abbreviations: N/n, counts; nEA, neuropsychiatric-specific electroacupuncture; PRO, patient-reported outcomes; sEA, sham electroacupuncture; T1, baseline; T2, ~5 weeks after baseline; T3, ~10 weeks after baseline; T4, ~4 weeks after treatment completion.  ^a^ Responders for PROs were defined as symptom improvement that has achieved minimal clinically important differences, and for objective cognition by achieving clinically significant improvement in at least 1 cognitive domain(s), achieving reliable change index (RCI<1.96). RCI for each cognitive domain were calculated by subtracting the raw scores (outcome measures) at follow-up timepoints from T1 scores, divided by the standard error of difference estimated from the T2-T1 change in the sEA group, in order to account of practice effects. RCIs for all domains have been adjusted such that a positive RCI indicates an improvement for the measured domain from baseline, while a negative RCI represents a decline from baseline. | | | | | | |

**Table S8: Plasma biomarkers descriptive statistics.**

|  | **EA Treatment** | **T1** | **T2** | **T3** | **T4** |
| --- | --- | --- | --- | --- | --- |
| Plasma biomarkers^a^, median (Q1, Q3) |  |  |  |  |  |
| BDNF, pg/mL | sEA | 1191  (719, 2706) | 2812  (647, 3326) | 2069  (1511, 4104) | 1350  (891, 2404) |
|  | nEA | 1911  (1044, 4107) | 1285  (980, 3110) | 1970  (1367, 4381) | 2980  (1252, 4403) |
| TrkB, pg/mL | sEA | 785  (514, 918) | 827  (600, 891) | 803  (672, 920) | 787  (664, 880) |
|  | nEA | 894  (547, 1077) | 762  (468, 988) | 719  (527, 764) | 608  (469, 801) |
| CXCL13, pg/mL | sEA | 131  (97.6, 165) | 132  (115, 149) | 127  (105, 166) | 119  (98.5, 160) |
|  | nEA | 131  (80.2, 190) | 160  (117, 206) | 171  (94.2, 216) | 110  (87.0, 210) |
| IL-1α, pg/mL | sEA | 8.97  (7.46, 9.79) | 9.51  (7.23, 10.6) | 10.1  (8.9, 10.8) | 10.1  (8.8, 11.4) |
|  | nEA | 9.82  (8.64, 10.6) | 9.42  (8.64, 10.6) | 9.48  (7.46, 9.87) | 9.64  (7.96, 10.1) |
| IL-6, pg/mL | sEA | 0  (0, 6.24) | 0  (0, 6.78) | 0  (0, 5.22) | 0  (0, 5.74) |
|  | nEA | 0  (0, 2.95) | 0  (0, 2.60) | 0  (0, 0) | 0  (0, 2.29) |
| IL-8, pg/mL | sEA | 6.79  (2.79, 9.84) | 6.24  (4.80, 9.80) | 5.28  (3.58, 8.54) | 4.14  (0, 7.04) |
|  | nEA | 6.90  (0.94, 7.79) | 2.27  (0, 12.8) | 0  (0, 7.74) | 3.66  (0, 10.3) |
| IL-10, pg/mL | sEA | 0.880  (0.420, 1.19) | 0.710  (0.360, 1.34) | 0.985  (0.675, 1.63) | 0.800  (0.425, 1.30) |
|  | nEA | 0.875  (0.552, 1.33) | 1.01  (0.705, 1.63) | 0.895  (0.71, 1.58) | 0.995  (0.785, 1.40) |
| IL-17A, pg/mL | sEA | 0  (0, 3.69) | 0  (0, 4.70) | 2.45  (0, 4.66) | 0.74  (0, 3.69) |
|  | nEA | 2.22  (0, 2.51) | 2.51  (0, 3.62) | 1.96  (0, 2.51) | 1.96  (0, 2.51) |
| IL-18, pg/mL | sEA | 194  (159, 277) | 197  (138, 299) | 225  (156, 274) | 235  (172, 312) |
|  | nEA | 241  (198, 320) | 282  (192, 336) | 280  (223, 338) | 256  (197, 356) |
| IL-21, pg/mL | sEA | 386  (278, 428) | 446  (301, 462) | 439  (392, 487) | 440  (382, 490) |
|  | nEA | 432  (397, 508) | 435  (389, 459) | 426  (333, 472) | 424  (369, 463) |
| IL-33, pg/mL | sEA | 0  (0, 0) | 0  (0, 0) | 0  (0, 0) | 0  (0, 0) |
|  | nEA | 0  (0, 0) | 0  (0, 2.32) | 0  (0, 0) | 0  (0, 4.54) |
| MCP-1, pg/mL | sEA | 164  (133, 230) | 177  (114, 205) | 172  (119, 215) | 158  (120, 213) |
|  | nEA | 175  (124, 235) | 205  (138, 247) | 143  (114, 171) | 159  (125, 216) |
| MIP-1α, pg/mL | sEA | 0  (0, 2.32) | 2.73  (0, 12.8) | 0.98  (0, 11.7) | 0  (0, 9.90) |
|  | nEA | 0  (0, 3.42) | 0  (0, 6.09) | 0  (0, 2.06) | 0  (0, 0) |
| RANTES, pg/mL | sEA | 1321  (835, 1771) | 1404  (879, 1744) | 1706  (1576, 2269) | 1836  (1489, 2487) |
|  | nEA | 1968  (1372, 4678) | 1488  (1209, 3214) | 1629  (1337, 4202) | 1837  (1260, 5704) |
| TNF-α, pg/mL | sEA | 9.78  (5.85, 11.9) | 6.83  (4.86, 14.3) | 6.83  (4.36, 12.8) | 8.36  (5.85, 12.8) |
|  | nEA | 10.2  (5.85, 16.2) | 10.2  (0, 13.0) | 11.5  (9.78, 14.3) | 11.5  (6.21, 14.3) |
| Abbreviations: BDNF, brain-derived neurotrophic factor; CXCL13, chemokine (C-X-C motif) ligand 13; IL, interleukin; MCP-1, monocyte chemoattractant protein 1; nEA, neuropsychiatric-specific electroacupuncture; Q1, quartile 1; Q3, quartile 3; RANTES, regulated on activation, normal T-cell expressed and secreted; sham electroacupuncture; TrkB, tropomyosin receptor kinase B.  ^a^ IL-1β, IL-4, and IL-23 were excluded as > 80% of values were below the lower limit of quantification. | | | | | |

**Table S9: Changes in plasma biomarkers from baseline to T3 and T4.**

| **Plasma biomarkers^a^** | **Timepoints** | **Baseline-adjusted mean change** | | **Between group differences** |
| --- | --- | --- | --- | --- |
|  |  | **Effect size^b,c^ (95% CI)** | | **Cohen’s *d*^b,d^** |
|  |  | **nEA** | **sEA** | **nEA vs sEA** |
| BDNF | T3 – T1 | -0.013 (-0.713, 0.688) | 0.511 (-0.165, 1.187) | -0.508 |
|  | T4 – T1 | 0.226 (-0.471, 0.924) | -0.057 (-0.733, 0.619) | 0.589 |
| TrkB | T3 – T1 | -0.127 (-0.898, 0.644) | 0.102 (-0.649, 0.852) | -0.346 |
|  | T4 – T1 | -0.461 (-1.232, 0.310) | 0.207 (-0.544, 0.958) | ***-0.882**** |
| CXCL13 | T3 – T1 | 0.032 (-0.829, 0.893) | -0.014 (-0.854, 0.827) | 0.050 |
|  | T4 – T1 | -0.057 (-0.918, 0.804) | -0.462 (-1.302, 0.379) | 0.441 |
| IL-1α | T3 – T1 | -0.001 (-0.884, 0.882) | 0.135 (-0.727, 0.998) | -0.165 |
|  | T4 – T1 | 0.008 (-0.875, 0.891) | -0.323 (-1.186, 0.539) | 0.332 |
| IL-6 | T3 – T1 | -0.356 (-1.033, 0.321) | 0.038 (-0.627, 0.704) | -0.600 |
|  | T4 – T1 | -0.073 (-0.750, 0.605) | -0.206 (-0.871, 0.459) | 0.144 |
| IL-8 | T3 – T1 | -0.593 (-1.304, 0.118) | 0.183 (-0.509, 0.874) | ***-1.132**** |
|  | T4 – T1 | -0.304 (-1.015, 0.407) | -0.309 (-1.001, 0.382) | -0.087 |
| IL-10 | T3 – T1 | 0.083 (-0.629, 0.795) | 0.194 (-0.500, 0.888) | -0.013 |
|  | T4 – T1 | 0.087 (-0.625, 0.799) | 0.111 (-0.583, 0.805) | 0.102 |
| IL-17A | T3 – T1 | -0.095 (-0.871, 0.681) | 0.271 (-0.491, 1.033) | -0.295 |
|  | T4 – T1 | -0.117 (-0.893, 0.659) | 0.020 (-0.742, 0.782) | -0.017 |
| IL-18 | T3 – T1 | 0.003 (-0.829, 0.834) | -0.029 (-0.840, 0.782) | 0.003 |
|  | T4 – T1 | -0.010 (-0.842, 0.822) | 0.067 (-0.744, 0.878) | -0.120 |
| IL-21 | T3 – T1 | -0.004 (-0.774, 0.767) | 0.480 (-0.271, 1.230) | -0.332 |
|  | T4 – T1 | -0.005 (-0.775, 0.766) | 0.068 (-0.683, 0.818) | 0.190 |
| IL-33 | T3 – T1 | 0.100 (-0.445, 0.645) | -0.109 (-0.635, 0.417) | 0.378 |
|  | T4 – T1 | 0.506 (-0.039, 1.051) | -0.250 (-0.776, 0.276) | ***1.341**** |
| MCP-1 | T3 – T1 | 0.203 (-0.628, 1.034) | 0.235 (-0.577, 1.047) | -0.073 |
|  | T4 – T1 | 0.262 (-0.569, 1.093) | -0.073 (-0.885, 0.739) | 0.342 |
| MIP-1α | T3 – T1 | 0.098 (-0.336, 0.531) | 0.361 (-0.057, 0.780) | -0.565 |
|  | T4 – T1 | -0.111 (-0.545, 0.322) | 0.246 (-0.173, 0.664) | -0.772 |
| RANTES | T3 – T1 | 0.270 (-0.480, 1.020) | 0.049 (-0.706, 0.805) | -0.036 |
|  | T4 – T1 | 0.303 (-0.447, 1.053) | 0.009 (-0.734, 0.751) | 0.057 |
| TNF-α | T3 – T1 | -0.227 (-1.090, 0.637) | 0.363 (-0.479, 1.204) | -0.667 |
|  | T4 – T1 | -0.035 (-0.899, 0.828) | -0.281 (-1.122, 0.561) | 0.242 |
| Abbreviations: BDNF, brain-derived neurotrophic factor; CI, confidence interval; CXCL13, chemokine (C-X-C motif) ligand 13; IL, interleukin; MCP-1, monocyte chemoattractant protein 1; nEA, neuropsychiatric-specific electroacupuncture; RANTES, regulated on activation, normal T-cell expressed and secreted; sham electroacupuncture; TrkB, tropomyosin receptor kinase B.  ^a^ IL-1β, IL-4, and IL-23 were excluded as > 80% of values were below the lower limit of quantification.  ^b^ Computed with log-transformed biomarker values.  ^c^ Glass’s Δ. Greater than 0 indicates an increase in biomarker levels from baseline while less than 0 indicates a reduction from baseline. Values of 0.2, 0.5, and 0.8 reflect small, medium, and large effect sizes, respectively.  ^d^ Cohen’s *d*. Greater than 0 indicates a larger increase in biomarker levels for nEA while less than 0 indicates a larger decrease for nEA, relative to sEA. Values of 0.2, 0.5, and 0.8 reflect small, medium, and large effect sizes, respectively.  * p < 0.05 for Cohen’s d. | | | | |

**Table S10: Pearson’s correlation analysis of gray and white matter and hippocampal metrics with cognitive domains and quality of life at T3, stratified by treatment group.**

| **Health outcomes^a^** | **Correlation (*p-value*)** | | | | | | | |
| --- | --- | --- | --- | --- | --- | --- | --- | --- |
|  | **nEA (N=12)** | | | | **sEA (N=11)** | | | |
|  | **Gray Matter Volume** | **White Matter Volume** | **Hippocampal Volume** | **Hippocampal Intensity** | **Gray Matter Volume** | **White Matter Volume** | **Hippocampal Volume** | **Hippocampal Intensity** |
| Attention | 0.686 *(0.020) | 0.568 (0.068) | 0.175 (0.607) | -0.155 (0.649) | 0.381 (0.222) | -0.115 (0.721) | 0.392 (0.208) | 0.024 (0.940) |
| Response | 0.214 (0.528) | -0.031 (0.928) | -0.194 (0.568) | -0.177 (0.602) | 0.623 *(0.030) | -0.125 (0.698) | 0.375 (0.230) | -0.061(0.851) |
| Executive function | 0.401 (0.222) | 0.396 (0.228) | 0.683 *(0.020) | 0.092 (0.787) | 0.001 (0.978) | -0.397 (0.201) | 0.047 (0.884) | -0.042 (0.896) |
| Quality of life | 0.228 (0.499) | -0.345 (0.299) | -0.091 (0.791) | 0.664 *(0.026) | -0.298 (0.347) | 0.359 (0.252) | -0.249 (0.435) | 0.0169 (0.958) |
| Abbreviations: EA, electroacupuncture; N, counts; nEA, neuropsychiatric-specific electroacupuncture; sEA, sham electroacupuncture.  ^a^ Health outcomes were standardized so that higher scores represent better outcomes.  * *p* < 0.05. | | | | | | | | |

**Table S11: Associations between changes in brain connectivity and cognitive measures of memory and attention.**

| **Cognitive measure** | **Seed** | **Cluster** | **Cluster Size (k)** | **nEA > sEA**  **(T-statistic, p-value)^a^** | **Peak Coordinates**  **(x, y, z)** |
| --- | --- | --- | --- | --- | --- |
| Memory | Default Mode Network | Left Superior Frontal Gyrus | 73 | T = 8.65, p = 0.005 | -20, 28, 58 |
| Memory | Dorsal Attention Network | Left Primary Somatosensory Cortex | 69 | T = -6.98, p = 0.008 | -46, -20, 24 |
| Memory | Right Frontoparietal Network | Left Inferior Frontal Gyrus | 61 | T = -6.65, p = 0.01 | -38, 04, 24 |
| Response speed | Dorsal Attention Network | Left Supramarginal Gyrus | 84 | T = -7.18, p = 0.002 | -54, -42, 38 |
| Response speed | Right Frontoparietal Network | Left Supramarginal Gyrus / Primary Somatosensory Cortex | 147 | T = -5.95, p < 0.001 | -52, -26, 46 |
| Abbreviations: nEA, neuropsychiatric-specific electroacupuncture; sEA, sham electroacupuncture.  ^a^ Group-level differences between nEA and sEA where changes in brain connectivity are differentially correlated with improvements in cognitive scores between the two groups (voxel-wise p < 0.001; cluster-wise FWE-corrected p < 0.05). | | | | | |

**Table S12: Patient acceptance of EA and treatment blinding outcomes.**

| **Feasibility outcomes** | **nEA (N=14)** | **sEA (N=16)** | **Total (N=30)** |
| --- | --- | --- | --- |
| Patient acceptance, n (%) |  |  |  |
| Satisfied with EA | 14 (100%) | 16 (100%) | 30 (100%) |
| Perceived benefit from EA | 12 (85.7%) | 13 (81.3%) | 25 (83.3%) |
| Perceived effectiveness in managing symptoms | 12 (85.7%) | 31 (75.0%) | 24 (80.0%) |
| Worthwhile to participate in EA trial | 14 (100%) | 16 (100%) | 30 (100%) |
| Will recommend EA to others | 13 (92.9%) | 16 (100%) | 29 (96.7%) |
| Will consider EA again outside of a trial | 13 (92.9%) | 15 (93.8%) | 28 (93.3%) |
| Overall experience with EA, n (%) |  |  |  |
| Better than expected | 7 (50.0%) | 11 (68.8%) | 18 (60.0%) |
| Same as expected | 6 (42.9%) | 5 (31.3%) | 11 (36.7%) |
| Worse than expected | 1 (7.1%) | 0 (0%) | 1 (3.3%) |
| Reasons for liking EA trial^a^, n (%) |  |  |  |
| Relaxation | 9 (64.3%) | 9 (56.3%) | 18 (60.0%) |
| Symptom improvement | 2 (14.3%) | 3 (18.8%) | 5 (16.7%) |
| Other health benefits | 2 (14.3%) | 1 (6.3%) | 3 (10.0%) |
| Experience with acupuncturist | 1 (7.1%) | 1 (6.3%) | 2 (6.7%) |
| Participating in a research study | 1 (7.1%) | 1 (6.3%) | 2 (6.7%) |
| Reasons for disliking EA trial^a^, n (%) |  |  |  |
| Expected adverse events | 9 (64.3%) | 7 (43.8%) | 16 (53.3%) |
| Transportation | 0 (0%) | 3 (18.8%) | 3 (10.0%) |
| Treatment group guesses, n (%) |  |  |  |
| Guess nEA | 7 (50.0%) | 6 (37.5%) | 13 (43.3%) |
| Guess sEA | 2 (14.3%) | 5 (31.3%) | 7 (23.3%) |
| Not sure | 5 (35.7%) | 5 (31.3%) | 10 (33.3%) |
| Abbreviations: EA, electroacupuncture; N/n, counts; nEA, neuropsychiatric-specific electroacupuncture; sEA, sham electroacupuncture.  ^a^ Free-response question. | | | |
