# Supplementary material for: Electroacupuncture improves cognitive function and neuropsychiatric symptoms in breast cancer survivors: a pilot randomized controlled trial
Source: J Natl Cancer Inst. Author manuscript; Available in PMC 2026 Jun 22. (PMC13285686; doi:10.1093/jnci/djag096)
Supplement: Supplementary Methods [file NIHMS2179458-supplement-Supplementary_Methods.docx]

Supplementary Methods

***Electroacupuncture Improves Cognition and Distress Symptoms in Breast Cancer Survivors: A Pilot RCT***

Contents

[Trial design 2](#_Toc214309646)

[Participants 2](#_Toc214309647)

[Randomization, Allocation Concealment, and Blinding 2](#_Toc214309648)

[Intervention 3](#_Toc214309649)

[Data Collection 3](#_Toc214309650)

[Objective Cognition 4](#_Toc214309651)

[Self-perceived Cognition 4](#_Toc214309652)

[Fatigue 4](#_Toc214309653)

[Psychological Distress, Insomnia, and Quality of Life 5](#_Toc214309654)

[Plasma Biomarkers 5](#_Toc214309655)

[Neuroimaging Procedures 6](#_Toc214309656)

[Safety 7](#_Toc214309657)

[Patient Acceptance and Blinding Assessment 7](#_Toc214309658)

[Study Endpoints 7](#_Toc214309659)

[Sample Size Calculation 7](#_Toc214309660)

[Statistical Analysis 7](#_Toc214309661)

[Neuroimaging Analysis 8](#_Toc214309662)

[STRICTA 2010 checklist 9](#_Toc214309663)

[CONSORT 2025 checklist 12](#_Toc214309664)

[References 16](#_Toc214309665)

# Trial design

This is a randomized, controlled, patient and assessor-blinded pilot trial conducted at University of California Irvine (UCI), the Chao Family Comprehensive Cancer Center (CFCCC), and the Susan Samueli Integrative Health Institute from 2022 to 2024 (ClinicalTrials.gov: NCT05283577). Participants were randomized to either one of the two arms: neuropsychiatric-specific EA (nEA) or sham EA (sEA). We obtained oncologists’ and institutional support from the CFCCC Breast Disease-Oriented Team (#UCI-21-33) and received ethics approval from the UCI Institutional Review Board (#2021-6732). The study protocol has been peer-reviewed and published^1^. This manuscript was prepared in accordance with the reporting guidelines for randomized trials (CONSORT 2025^2^) and its extension module for acupuncture studies (STRICTA 2010^3^).

# Participants

Participants fulfilled the following inclusion criteria: diagnosed with cancer and have received anti-cancer treatment, ≥18 years of age, life expectancy of ≥6 months, and complaints of one or more of the following symptoms: cognitive impairment, fatigue, insomnia and psychological distress. Patients are ineligible if they have severe needle phobia, severe psychiatric/mental disorders which would affect neurocognitive assessment, have pacemakers or other electronic implants, epilepsy, had received acupuncture treatment in the three months prior, or were breastfeeding, pregnant, or planning to get pregnant during the study period. Participants with magnetic metal implants, severe claustrophobia, or non-removable metallic dental devices were ineligible for neuroimaging but remained eligible for EA interventions.

We determined potential participants primarily from medical record screening and physician referrals at the CFCCC. Other sources included the UCI Health Honest Broker System, the UCI Institute for Memory Impairments and Neurological Disorders (UCI MIND) Consent-2-Contact Registry of patients interested in clinical research participation^4^, and the community in partnership with Breast Cancer Angels, a local breast cancer advocacy organization. All participants were identified after screening by a research coordinator and provided written informed consent prior to participation.

# Randomization, Allocation Concealment, and Blinding

Treatment allocation in the ratio of 1:1 was randomly generated in random blocks of four or six prior to study initiation and held by a team member who is not involved in participant recruitment or intervention implementation. Allocation concealment was ensured by the same team member who distributed the treatment label sequentially on an electronic platform upon confirmation of participant eligibility and obtainment of informed consent. Access to the platform is restricted to this team member and acupuncturists, and only the acupuncturists were aware of the true treatment allocation.

# Intervention

Regardless of treatment allocation, participants received intervention with ten weekly EA sessions, administered by licensed acupuncturists, over a period of 10-12 weeks. The 2-week buffer was considered in the event of unexpected, missed treatment visits such as COVID-19 exposure. During each session, participants lie in a supine position with needles inserted at depth of 9-24 mm and angle of 15-90 degrees depending on the location of the needle. Electrodes from the EA unit ES-160 (Ito Co. Ltd., Tokyo, Japan) were attached to each needle handle following needle insertion, and the EA stimulation lasted for 30 minutes with a continuous wave of 2 Hz. The EA intensity was adjusted based on individual participants’ threshold and sensitivity. Each EA session lasted between 30 minutes to an hour.

- *Neuropsychiatric-specific EA (nEA):* Participants in the nEA group received EA at disease-related acupoints selected by a licensed acupuncturist with over ten years of experience. The acupoints were decided by acupuncturists and further rationalized based on published scientific evidence supporting their anti-inflammatory, stress-modulating, and neurogenic properties, as well as clinical data demonstrating their efficacy in alleviating cognitive impairment, fatigue, and distress symptoms in both cancer and non-cancer populations. Specific details of the protocol can be found in Table S1.
- *Sham EA (sEA):* Participants in the sEA group received EA at non-disease-related acupoints, at an EA stimulation and intensity indistinguishable from the nEA intervention to minimize risk of unblinding.

# Data Collection

We collected data at three timepoints for each participant: baseline (pre-treatment, T1), mid-treatment (~5 weeks from baseline, T2), end of treatment (~10 weeks from baseline, T3), and ~4 weeks after treatment (~14 weeks from baseline, i.e. ~4 weeks post-treatment, T4) (Figure S1). Sociodemographic characteristics and breast cancer-related information were obtained from participants at baseline and supplemented with electronic medical records data as necessary. At each timepoint, participants completed four questionnaires capturing patient-reported outcomes (PROs) on neuropsychiatric symptoms, quality of life and health utility, a cognitive battery of neuropsychological tests to evaluate cognitive function in domains of attention, memory, response speed, executive function, and multitasking, as well as 10 mL of peripheral blood for assessment of biomarkers of neurogenesis and inflammation. Neuroimaging was performed at baseline and within one week after treatment completion (T3) for neuroimaging-eligible patients. After completing all treatment sessions, participants were provided with a survey to evaluate their acceptance towards the EA interventions they had received.

# Objective Cognition

The Cambridge Neuropsychological Test Automated Battery (CANTAB®) is a computerized objective assessment, administered on a tablet, to evaluate participants’ performance in cognitive domains of attention (rapid visual information processing), memory (paired associates learning), response speed (reaction time), executive function (spatial working memory), and multitasking (multitasking test)^5^. The collection of longitudinal data of these cognitive domains is in accordance with recommendations from the International Cognition and Cancer Task Force (ICCTF)^6^.

- *Outcome measures:* We selected key outcome measures for each domain in accordance with product company’s (Cambridge Cognition, Cambridge, England) recommendations. Table S2 provides a description of selected outcome measures.
- *Treatment responders:* Reliable change indices (RCI) for each cognitive domain were calculated by subtracting the raw scores (outcome measures) at follow-up timepoints from baseline (T1) scores, divided by the standard error of difference estimated from the T2-T1 change in the sEA group, in order to account of practice effects^7^. RCIs for all domains have been adjusted such that a positive RCI indicates an improvement for the measured domain from baseline, while a negative RCI represents a decline from baseline. Treatment responders are defined as participants reporting a clinically significant improvement (RCI > 1.96, <5% probability of improving by chance alone^8^) in ≥1 domain(s) at T3 and T4, after EA treatment and approximately four weeks later, respectively.

# Self-perceived Cognition

The Functional Assessment of Cancer Therapy—Cognitive Function version 3 (FACT-Cog) is a validated questionnaire administered for evaluating self-perceived cognitive function in cancer patients and survivors^9,10^.

- *Outcome measures:* The FACT-Cog total score (range: 0 to 148) was calculated by summing all 33 items with higher scores representing better self-perceived cognition.
- *Treatment responders:* Treatment responders comprised participants who achieved an improvement of ≥15.0 points in the FACT-Cog total score, which represents the minimal clinically important difference (MCID), from baseline to T3 and T4^11^.

# Fatigue

The Multidimensional Fatigue Symptom Inventory-Short Form (MFSI-SF) is a validated questionnaire administered for evaluating fatigue levels in cancer patients and survivors^12,13^.

- *Outcome measures:* The MFSI-SF total score (range: -24 to 96) was calculated by subtracting the vigor subscale score (6 items) from a sum score of 24 items from four subscales (general fatigue, physical fatigue, emotional fatigue, and mental fatigue), with higher score indicating worse fatigue symptoms.
- *Treatment responders:* Treatment responders comprised participants who reported a reduction in MFSI-SF total score by ≥10.79 points, which represents the MCID, from baseline to T3 and T4^13,14^.

# Psychological Distress, Insomnia, and Quality of Life

The European Organization for Research and Treatment of Cancer Core Quality of Life (EORTC QLQ-C30) is a validated questionnaire administered in this study for evaluating psychological distress, insomnia, and quality of life in cancer patients and survivors^15^.

- *Outcome measures:* The outcome measures (range: 0 to 100) of interest include emotional functioning (EF, 2 items) functional scale for evaluating psychological distress, insomnia (SL, 1 item) symptom scale, as well as global health status (GHS, 2 items) to measure quality of life. Higher scale scores represent higher response level, meaning that higher EF and GHS, and lower SL represent better outcomes^16^.
- *Treatment responders:* Treatment responders for each outcome was determined based on published pooled data of breast cancer populations reflecting at least a medium effect size of improvement in subscale scores [i.e., ≥0.5 standard deviation (SD)], being greater than the measurement error [i.e., standard error of the mean (SEM)], while also considering the minimal change achievable for an individual between two timepoints, whichever is larger^17,18^. Therefore, the MCID thresholds for the three subscales are as follows: an increase of ≥11.77 points in EF, an increase of ≥12.16 points in GHS, and a decrease of ≥33.33 points in SL^17,18^.

# Plasma Biomarkers

A 10-mL blood sample was drawn, stored in ethylenediaminetetraacetic (EDTA) acid tubes, and then centrifuged at 1,069 x *g* for 10 minutes at 4°C. The resulting plasma was aliquoted and stored at -80 °C freezer until analysis.

BDNF levels were measured from plasma sample that was diluted per manufacturer instructions. The quantification was performed using a commercially available enzyme-linked immunosorbent assay (ELISA) kit (Biosensis BEK-2211-1P/2P, Australia). Assays were conducted in duplicates following the manufacturer’s protocol, and an intra-assay coefficient of variation below 10% was considered acceptable. Concentrations were determined using a four-parameter logistic regression model and presented in pg/mL.

Plasma tropomyosin receptor kinase B (TrkB), and cytokine levels were quantified using the ProcartaPlex™ TrkB Simplex Kit (Thermo Fisher Scientific EPX010-12428-901, United States) and a custom multiplex (Thermo Fisher Scientific PPX-17-MXPRNHD, United States), respectively. Cytokines and chemokines assessed in the multiplex include C-X-C motif chemokine ligand 13 (CXCL13), interleukin (IL)-1α, IL-1β, IL-4, IL-6, IL-8, IL-10, IL-17A, IL-18, IL-21, IL-23, IL-33, monocyte chemoattractant protein-1 (MCP-1), macrophage inflammatory protein-1 alpha (MIP-1α), regulated on activation, normal T cell expressed and secreted (RANTES), and tumor necrosis factor-alpha (TNF-α). To minimize matrix effects, plasma samples for the multiplex were diluted four-fold with ProcartaPlex™ Platinum Assay Buffer (Thermo Fisher Scientific EPXP-11112-000, United States). Assays were conducted in duplicates following the manufacturer’s protocol. Concentrations were determined from standard curves generated by standards of known analyte concentrations and presented in pg/mL using the ProcartaPlex Analysis App.

Cytokines and chemokines with undetectable levels based on assay sensitivity were considered as 0 pg/ml. Biomarkers with > 80% of values that were below the lower limit of quantification were excluded from the final analysis^19^.

# Neuroimaging Procedures

All neuroimaging procedures were completed using a 3T Siemens Prisma scanner with a 32-channel head coil at the UCI Facility for Imaging and Brain Research. Following ICCTF’s recommendations, we acquired high-resolution structural, diffusion, FLAIR, and function MRI scans using harmonized parameters (ADNI-3 MRI protocol). High-resolution T1-weighted anatomical images were acquired using an accelerated sagittal MPRAGE sequence (TR = 2300 ms, TE = 2.98 ms, TI = 900 ms, flip angle = 9°, voxel size = 1.0 mm³ isotropic, matrix = 256 × 240, GRAPPA factor = 2). Resting-state fMRI data were collected using a multiband echo-planar imaging sequence (TR = 607 ms, TE = 32 ms, flip angle = 50°, voxel size = 2.5 mm³ isotropic, matrix = 88 × 88, 64 slices, multiband factor = 8) while participants rested with eyes open. T2-weighted images were acquired using a 3D sagittal SPACE (variable-flip-angle turbo spin-echo) sequence with the following parameters: TR = 3200 ms, TE = 564 ms, flip angle = 120° (variable), voxel size = 1.0 mm³ isotropic, matrix = 240 × 240, parallel imaging (GRAPPA) = 2.

These sequences allow us to evaluate structural quantification, white matter integrity (mean diffusivity, fractional anisotropy, radial and axial diffusivities) and functional connectivity of the neural networks^20^.

fMRI data were preprocessed using **fMRIPrep** (v23.2.0)^21^. Preprocessing included skull stripping, rigid-body motion correction, and nonlinear registration of functional images to the MNI152 standard template. **Slice-timing correction was omitted because data were acquired with a short TR (607 ms) and multiband acceleration (factor = 8), minimizing temporal offsets across slices and rendering STC unnecessary.** To reduce equilibration and T1 stabilization effects, the first 10 volumes of each run were discarded. Functional images were spatially smoothed with a 6-mm FWHM Gaussian kernel using AFNI’s 3dBlurInMask to improve signal-to-noise ratio^22^. Framewise displacement (FD) was calculated for each run, with volumes exceeding 0.5 mm flagged for motion censoring^23,24^. Participants were excluded if more than 30% of volumes exceeded this FD threshold; only 1 participant met this criterion. Additional nuisance regressors, including six rigid-body motion parameters and aCompCor components, were incorporated to further reduce head motion and physiological noise prior to functional connectivity analyses.

# Safety

Participants were monitored for adverse events (AEs) during and after each treatment session. Severity of symptoms were graded according to the Common Terminology Criteria for Adverse Events (CTCAE) version 5.0^25^.

# Patient Acceptance and Blinding Assessment

After ten EA sessions, participants were asked to guess their group allocation (treatment or control or "not sure"). They were also asked if they were satisfied, felt they benefited, and would consider EA again outside of a trial.

# Study Endpoints

To evaluate preliminary efficacy of the regimen, we utilize co-primary endpoints comprising within-group changes from baseline to end-of-treatment (T3) and post-treatment (T4) across ten independent symptom domains encompassing objective cognitive domains (attention, memory, response speed, executive function, multitasking) and patient-reported outcomes (subjective cognition, fatigue, distress, insomnia, quality of life). To maintain integrity of the findings, we applied the Benjamini-Hochberg method to account for multiple testing across the symptom domains at each timepoint.

Secondary endpoints evaluated between-group changes, compared proportions of treatment responders, and comparing the proportions of AEs between nEA and sEA. Responders for PROs were defined as symptom improvement that has achieved minimal clinically important differences (MCID)^11,13,14,17,18^, and for objective cognition by achieving clinically significant improvement in at least 1 cognitive domain(s), achieving reliable change index (RCI<1.96) at T3 and T4^7,8^. Calculated between-group effect sizes will inform future sample size requirements for a future, larger-scale definitive RCT.

Exploratory endpoints included the pre-post changes plasma and neuroimaging biomarkers, and their correlations with health outcomes, pre-post changes in health utility, safety and feasibility.

# Sample Size Calculation

As this is a preliminary efficacy pilot and existing literature lacks precise variance estimates for the differences in neuropsychiatric outcomes between nEA and sEA, the sample size was not determined based on a formal power calculation for hypothesis testing. Instead, the study aims to provide the necessary parameters to inform a future definitive trial. Following published guidance for sample size recommendations in pilot trials,^26–29^ a minimum of 30 evaluable participants completing all research procedures, 15 in each arm, should be recruited. Our final sample size, after accounting for an assumed dropout rate of 10%, is 34 participants.

# Statistical Analysis

Participant characteristics were compared between treatment groups with summary statistics of counts and proportions for categorical variables, as well as means and standard deviations (SD) for continuous variables, or median and interquartile ranges for non-normal outcomes.

For the primary endpoint analysis for each health outcome measure, we computed group-specific mean changes and treatment effect sizes using Glass's Δ, with 95% confidence intervals (CIs), adjusted for baseline variability (small [0.2], medium [0.5], large [0.8]^30,31^) using linear mixed models with fixed effects for baseline outcome, group, time (modeled as a categorical variable) and group-time interaction, and random intercepts for participants. At each data collection timepoint, multiple testing of health outcomes was controlled via the Benjamini-Hochberg method^32^, with statistical significance set at *p-adjusted* < 0.05. All observations and participants’ data were included in the model to facilitate intention-to-treat analysis. To compare changes in continuous outcome measures between the groups, we calculated Cohen's *d* to quantify the effect size for change observed with nEA intervention relative to sEA using the fitted linear mixed models as previously described. Positive values for Cohen’s *d* (small [0.2], medium [0.5], large [0.8]^30,31^) indicate greater improvements in nEA compared to sEA*.* A sensitivity analysis was conducted to adjust for placebo effect by including participants’ guesses of treatment allocation (“treatment group”, “control group”, “not sure”) as a confounder in the model. The proportions of treatment responders (defined separately for each health outcome and for the combined summation of all outcomes) were descriptively summarized for comparison between the groups. A difference of at least 10% for each outcome is considered clinically important. Similar analyses were conducted for safety and feasibility outcomes.

Changes in plasma biomarkers (post-intervention minus baseline) were also modeled with the same model specification with linear mixed models. Biomarker values were normalized using a natural log transformation to correct for positive skewness. Subsequently, the log-transformed values were standardized into z-scores to enable direct comparison and scaling of different measures, and to facilitate computation of effect sizes reflecting pre-to-post treatment changes (Glass's Δ). Undetectable biomarker levels were imputed as 0.001 to facilitate log transformation in modeling and correlation analysis. Batch-to-batch variability was accounted for as a random slope effect. For each treatment group, we used a Spearman correlation (*rho*) matrix to examine the relationship between relative changes (absolute change divided by baseline) in plasma biomarkers and health outcomes, from baseline to T3 (end-of-treatment). For this analysis, we standardized the changes in health outcomes so that positive values correspond to an improved outcome from baseline.

Exploratory analysis of EQ-5D health utility involved descriptively summarizing metrics across timepoints for each treatment group and computing Cohen's *d* to quantify between-group effect sizes. Safety and patient acceptance outcomes were analyzed descriptively.

All analyses were two-tailed, tested at 5% significance level, and performed with R version 4.4.1^33^ unless otherwise specified.

# Neuroimaging Analysis

The structural (T1-weighted and T2-weighted) MRI images were analyzed with FSL^34^ (FMIRB Software Library, Oxford University). In both structural images, the skulls were first removed with FSL’s BET tool, and then the T1- and T2-weighted images were aligned with the FLIRT tool. Gray matter, white matter and cerebrospinal fluid were segmented using the FAST tool and their respective volumes were calculated using partial volumes estimates with fslstats. The hippocampus was segmented using the FIRST tool and again the volume calculated with fslstats. A Pearson’s correlation analysis was performed comparing the neurocognitive scores with the imaging metrics for each treatment group.

Resting-state fMRI data were analyzed using group-level independent component analysis (ICA) with FSL’s MELODIC, applying temporal concatenation across participants and fixing the dimensionality to 20 components to capture canonical resting-state networks. ICA spatial maps were matched to template maps from Beckmann et al.^35^ and Smith et al.^36^ using spatial correlation, which allowed the identification of the default mode (DMN), sensorimotor (SMN), left and right frontoparietal (FPN), and dorsal attention (DAN) networks. Because no distinct salience network (SLN) component was identified from the ICA decomposition, we additionally defined the left and right insular cortex (IC) as seed regions based on the Harvard–Oxford cortical atlas to assess SLN-related connectivity patterns.

Functional connectivity (FC) analyses were carried out using the CONN toolbox (v22.a) implemented in MATLAB/SPM12. Seed-to-whole-brain analyses were performed using the ICA-derived network maps, as well as the left and right insula, as seed regions. At the second-level analysis, group differences in FC changes were modeled with mean-centered change in objective cognition measures (memory, attention, response speed) as covariates of interest, as these measures were greatly improved after nEA compared to sEA. Age was matched between the nEA and sEA groups in the fMRI subsample and was not entered as a covariate due to limited sample size.

Outliers for each cognitive outcome were identified using Grubbs’ test and excluded from further analysis. Additionally, one participant was excluded due to excessive head motion, defined as >30% of volumes with framewise displacement (FD) > 0.5 mm. After these exclusions, the final sample sizes were 12 nEA and 9 sEA participants for the memory analysis, 12 nEA and 10 sEA for attention, and 11 nEA and 10 sEA for response speed. Voxel-wise statistical significance was set at *p* < 0.001 (uncorrected), with a cluster-level family-wise error (FWE) correction at *p* < 0.05 to adjust for multiple comparisons. This approach allowed us to assess whether neural correlates of cognitive improvement varied as a function of treatment group.

# STRICTA 2010 checklist

| **Item** | **Detail** |
| --- | --- |
| **1. Acupuncture rationale** | 1a) Style of acupuncture – Traditional Chinese Medicine |
|  | 1b) Reasoning for treatment provided, based on historical context, literature sources, and/or consensus methods, with references where appropriate – Expert group consensus based on historical context |
|  | 1c) Extent to which treatment was varied – No variation |
| **2. Details of needling** | 2a) Number of needle insertions per subject per session (mean and range where relevant) – nEA (24) vs sEA (14) |
|  | 2b) Names (or location if no standard name) of points used (uni/bilateral) – See Table S1. |
|  | 2c) Depth of insertion, based on a specified unit of measurement, or on a particular tissue level  nEA 9–24 mm, 15–30° at GV24, EX-HN1, and GV20, and 90° at other body points. 2Hz individually adjusted EA intensity.  sEA a superficial insertion, 2Hz minimal EA stimulation with the same EA sounds |
|  | 2d) Response sought (e.g. *de qi* or muscle twitch response) – nEA with de qi, sEA without de qi, |
|  | 2e) Needle stimulation (e.g. manual, electrical) – EA |
|  | 2f) Needle retention time – 30 min |
|  | 2g) Needle type (diameter, length, and manufacturer or material) – size 0.20x25mm, AcuBEST(South El Monte, USA) |
| **3. Treatment regimen** | 3a) Number of treatment sessions – 10 sessions |
|  | 3b) Frequency and duration of treatment sessions – once per week, 30 minutes per session |
| **4. Other components of treatment** | 4a) Details of other interventions administered to the acupuncture group (e.g. moxibustion, cupping, herbs, exercises, lifestyle advice) – N/A |
|  | 4b) Setting and context of treatment, including instructions to practitioners, and information and explanations to patients – a randomized, sham-controlled, patient and assessor-blinded trial |
| **5. Practitioner background** | 5) Description of participating acupuncturists (qualification or professional affiliation, years in acupuncture practice, other relevant experience) – Two California licensed acupuncturists with PhD degrees with more than 20 years of acupuncture practice. |
| **6. Control or comparator interventions** | 6a) Rationale for the control or comparator in the context of the research question, with sources that justify this choice – non-disease related acupoints |
|  | 6b) Precise description of the control or comparator. If sham acupuncture or any other type of acupuncture-like control is used, provide details as for Items 1 to 3 above – Mentioned in 1 to 3 |

# CONSORT 2025 checklist

| **Section/topic** | **No** | **CONSORT 2025 checklist item description** | **Y/N (page no. for methods)** |
| --- | --- | --- | --- |
| **Title and abstract** | | |  |
| Title and structured abstract | 1a | Identification as a randomised trial | Y |
|  | 1b | Structured summary of the trial design, methods, results, and conclusions | N |
| **Open science** | | |  |
| Trial registration | 2 | Name of trial registry, identifying number (with URL) and date of registration | Y |
| Protocol and statistical analysis plan | 3 | Where the trial protocol and statistical analysis plan can be accessed | Y |
| Data sharing | 4 | Where and how the individual de-identified participant data (including data dictionary), statistical code and any other materials can be accessed | N |
| Funding and conflicts of interest | 5a | Sources of funding and other support (eg, supply of drugs), and role of funders in the design, conduct, analysis and reporting of the trial | Y |
|  | 5b | Financial and other conflicts of interest of the manuscript authors | Y |
| **Introduction** | | |  |
| Background and rationale | 6 | Scientific background and rationale | Y |
| Objectives | 7 | Specific objectives related to benefits and harms | Y |
| **Methods** | | |  |
| Patient and public involvement | 8 | Details of patient or public involvement in the design, conduct and reporting of the trial | N |
| Trial design | 9 | Description of trial design including type of trial (eg, parallel group, crossover), allocation ratio, and framework (eg, superiority, equivalence, non-inferiority, exploratory) | Y (2) |
| Changes to trial protocol | 10 | Important changes to the trial after it commenced including any outcomes or analyses that were not prespecified, with reason | N |
| Trial setting | 11 | Settings (eg, community, hospital) and locations (eg, countries, sites) where the trial was conducted | Y (2) |
| Eligibility criteria | 12a | Eligibility criteria for participants | Y (2) |
|  | 12b | If applicable, eligibility criteria for sites and for individuals delivering the interventions (eg, surgeons, physiotherapists) | N |
| Intervention and comparator | 13 | Intervention and comparator with sufficient details to allow replication. If relevant, where additional materials describing the intervention and comparator (eg, intervention manual) can be accessed | Y (3) |
| Outcomes | 14 | Prespecified primary and secondary outcomes, including the specific measurement variable (eg, systolic blood pressure), analysis metric (eg, change from baseline, final value, time to event), method of aggregation (eg, median, proportion), and time point for each outcome | Y (7) |
| Harms | 15 | How harms were defined and assessed (eg, systematically, non-systematically) | Y (7) |
| Sample size | 16a | How sample size was determined, including all assumptions supporting the sample size calculation | Y (8) |
|  | 16b | Explanation of any interim analyses and stopping guidelines | N |
| Randomisation: |  |  |  |
| Sequence generation | 17a | Who generated the random allocation sequence and the method used | Y (2) |
|  | 17b | Type of randomisation and details of any restriction (eg, stratification, blocking and block size) | Y (2) |
| Allocation concealment mechanism | 18 | Mechanism used to implement the random allocation sequence (eg, central computer/telephone; sequentially numbered, opaque, sealed containers), describing any steps to conceal the sequence until interventions were assigned | Y (2) |
| Implementation | 19 | Whether the personnel who enrolled and those who assigned participants to the interventions had access to the random allocation sequence | Y (2) |
| Blinding | 20a | Who was blinded after assignment to interventions (eg, participants, care providers, outcome assessors, data analysts) | Y (2) |
|  | 20b | If blinded, how blinding was achieved and description of the similarity of interventions | Y (2) |
| Statistical methods | 21a | Statistical methods used to compare groups for primary and secondary outcomes, including harms | Y (8) |
|  | 21b | Definition of who is included in each analysis (eg, all randomised participants), and in which group | Y (8) |
|  | 21c | How missing data were handled in the analysis | N |
|  | 21d | Methods for any additional analyses (eg, subgroup and sensitivity analyses), distinguishing prespecified from post hoc | Y (8) |
| **Results** | | |  |
| Participant flow, including flow diagram | 22a | For each group, the numbers of participants who were randomly assigned, received intended intervention, and were analysed for the primary outcome | Y |
|  | 22b | For each group, losses and exclusions after randomisation, together with reasons | Y |
| Recruitment | 23a | Dates defining the periods of recruitment and follow-up for outcomes of benefits and harms | Y |
|  | 23b | If relevant, why the trial ended or was stopped | N |
| Intervention and comparator delivery | 24a | Intervention and comparator as they were actually administered (eg, where appropriate, who delivered the intervention/comparator, how participants adhered, whether they were delivered as intended (fidelity)) | Y |
|  | 24b | Concomitant care received during the trial for each group | N |
| Baseline data | 25 | A table showing baseline demographic and clinical characteristics for each group | Y |
| Numbers analysed,  outcomes and estimation | 26 | For each primary and secondary outcome, by group:  ● the number of participants included in the analysis  ● the number of participants with available data at the outcome time point  ● result for each group, and the estimated effect size and its precision (such as 95% confidence interval)  ● for binary outcomes, presentation of both absolute and relative effect size | Y |
| Harms | 27 | All harms or unintended events in each group | Y |
| Ancillary analyses | 28 | Any other analyses performed, including subgroup and sensitivity analyses, distinguishing pre-specified from post hoc | Y |
| **Discussion** | | |  |
| Interpretation | 29 | Interpretation consistent with results, balancing benefits and harms, and considering other relevant evidence | Y |
| Limitations | 30 | Trial limitations, addressing sources of potential bias, imprecision, generalisability, and, if relevant, multiplicity of analyses | Y |

# References

1. Xie L, Ng DQ, Heshmatipour M, et al. Electroacupuncture for the management of symptom clusters in cancer patients and survivors (EAST). *BMC Complement Med Ther*. 2023;23(1). doi:10.1186/s12906-023-03926-9

2. Hopewell S, Chan AW, Collins GS, et al. CONSORT 2025 statement: Updated guideline for reporting randomised trials. *BMJ*. 2025;389. doi:10.1136/BMJ-2024-081123,

3. Macpherson H, Altman DG, Hammerschlag R, et al. Revised standards for reporting interventions in clinical trials of acupuncture (stricta): Extending the consort statement. *PLoS Med*. 2010;7(6). doi:10.1371/JOURNAL.PMED.1000261,

4. Grill JD, Hoang D, Gillen DL, et al. Constructing a Local Potential Participant Registry to Improve Alzheimer’s Disease Clinical Research Recruitment. *Journal of Alzheimer’s Disease*. 2018;63(3):1055-1063. doi:10.3233/JAD-180069

5. Chan A, Cheng I, Wang C, et al. Cognitive impairment in adolescent and young adult cancer patients: Pre-treatment findings of a longitudinal study. *Cancer Med*. 2023;12(4):4821-4831. doi:10.1002/cam4.5295

6. Wefel JS, Vardy J, Ahles T, Schagen SB. International Cognition and Cancer Task Force recommendations to harmonise studies of cognitive function in patients with cancer. *Lancet Oncol*. 2011;12(7):703-708. doi:10.1016/S1470-2045(10)70294-1

7. Maassen GH, Bossema E, Brand N. Reliable change and practice effects: Outcomes of various indices compared. *J Clin Exp Neuropsychol*. 2009;31(3):339-352. doi:10.1080/13803390802169059

8. Jacobson NS, Truax P. Clinical Significance: A Statistical Approach to Defining Meaningful Change in Psychotherapy Research. *J Consult Clin Psychol*. 1991;59(1):12-19. doi:10.1037/0022-006X.59.1.12

9. Cheung YT, Lim SR, Shwe M, Tan YP, Chan A. Psychometric properties and measurement equivalence of the english and chinese versions of the functional assessment of cancer therapy-cognitive in Asian patients with breast cancer. *Value in Health*. 2013;16(6):1001-1013. doi:10.1016/j.jval.2013.06.017

10. Cheung YT, Foo YL, Shwe M, et al. Minimal clinically important difference (MCID) for the functional assessment of cancer therapy: Cognitive function (FACT-Cog) in breast cancer patients. *J Clin Epidemiol*. 2014;67(7):811-820. doi:10.1016/j.jclinepi.2013.12.011

11. Bell ML, Dhillon HM, Bray VJ, Vardy JL. Important differences and meaningful changes for the Functional Assessment of Cancer Therapy-Cognitive Function (FACT-Cog). *J Patient Rep Outcomes*. 2018;2:48. doi:10.1186/S41687-018-0071-4

12. Chan A, Lew C, Wang XJ, et al. Psychometric properties and measurement equivalence of the Multidimensional Fatigue Syndrome Inventory- Short Form (MFSI-SF) amongst breast cancer and lymphoma patients in Singapore. *Health Qual Life Outcomes*. 2018;16(1). doi:10.1186/s12955-018-0846-6

13. Chan A, Yo TE, Wang XJ, et al. Minimal Clinically Important Difference of the Multidimensional Fatigue Symptom Inventory-Short Form (MFSI-SF) for Fatigue Worsening in Asian Breast Cancer Patients. *J Pain Symptom Manage*. 2018;55(3):992-997.e2. doi:10.1016/j.jpainsymman.2017.10.014

14. Li X, Liou KT, Chimonas S, et al. Addressing cancer-related fatigue through sleep: A secondary analysis of a randomized trial comparing acupuncture and cognitive behavioral therapy for insomnia. *Integr Med Res*. 2023;12(1). doi:10.1016/J.IMR.2023.100922

15. Aaronson NK, Ahmedzai S, Bergman B, et al. The European organization for research and treatment of cancer QLQ-C30: A quality-of-life instrument for use in international clinical trials in oncology. *J Natl Cancer Inst*. 1993;85(5):365-376. doi:10.1093/jnci/85.5.365

16. Fayers PM  Bjordal K, Groenvold M, Curran D, Bottomley A, on behalf of the EORTC Quality of Life Group. ANK. *EORTC QLQ-C30 Scoring Manual The EORTC QLQ-C30 Introduction*. Vol 30. European Organisation for Research and Treatment of Cancer, Brussels 2001; 2001. http://www.eortc.be/qol/files/scmanualqlq-c30.pdf

17. Musoro JZ, Coens C, Sprangers MAG, et al. Minimally important differences for interpreting EORTC QLQ-C30 change scores over time: A synthesis across 21 clinical trials involving nine different cancer types. *Eur J Cancer*. 2023;188. doi:10.1016/j.ejca.2023.04.027

18. Cocks K, Buchanan J. How scoring limits the usability of minimal important differences (MIDs) as responder definition (RD): an exemplary demonstration using EORTC QLQ-C30 subscales. *Quality of Life Research*. 2023;32(5). doi:10.1007/s11136-022-03181-4

19. Yap NY, Toh YL, Tan CJ, Acharya MM, Chan A. Relationship between cytokines and brain-derived neurotrophic factor (BDNF) in trajectories of cancer-related cognitive impairment. *Cytokine*. 2021;144. doi:10.1016/j.cyto.2021.155556

20. Deprez S, Kesler SR, Saykin AJ, Silverman DHS, De Ruiter MB, McDonald BC. International cognition and cancer task force recommendations for neuroimaging methods in the study of cognitive impairment in non-CNS cancer patients. *J Natl Cancer Inst*. *Oxford University Press*. 2018;110(3):223-231. doi:10.1093/jnci/djx285

21. Esteban O, Markiewicz CJ, Blair RW, et al. fMRIPrep: a robust preprocessing pipeline for functional MRI. *Nat Methods*. 2019;16(1):111-116. doi:10.1038/S41592-018-0235-4

22. Cox RW. AFNI: Software for analysis and visualization of functional magnetic resonance neuroimages. *Computers and Biomedical Research*. 1996;29(3):162-173. doi:10.1006/cbmr.1996.0014

23. Power JD, Mitra A, Laumann TO, Snyder AZ, Schlaggar BL, Petersen SE. Methods to detect, characterize, and remove motion artifact in resting state fMRI. *Neuroimage*. 2014;84:320-341. doi:10.1016/j.neuroimage.2013.08.048

24. Power JD, Barnes KA, Snyder AZ, Schlaggar BL, Petersen SE. Spurious but systematic correlations in functional connectivity MRI networks arise from subject motion. *Neuroimage*. 2012;59(3):2142-2154. doi:10.1016/j.neuroimage.2011.10.018

25. National Institutes of Health. Common Terminology Criteria for Adverse Events (CTCAE) version 5.0. NIH Publication. November 27, 2017. Accessed December 23, 2024. https://ctep.cancer.gov/protocolDevelopment/electronic_applications/docs/CTCAE_v5_Quick_Reference_8.5x11.pdf

26. Kieser M, Wassmer G. On the use of the upper confidence limit for the variance from a pilot sample for sample size determination. *Biometrical Journal*. 1996;38(8):941-949. doi:10.1002/bimj.4710380806

27. Browne RH. On the use of a pilot sample for sample size determination. *Stat Med*. 1995;14(17):1933-1940. doi:10.1002/sim.4780141709

28. Birkett MA, Day SJ. Internal pilot studies for estimating sample size. *Stat Med*. 1994;13(23-24):2455-2463. doi:10.1002/sim.4780132309

29. Desmond JE, Glover GH. Estimating sample size in functional MRI (fMRI) neuroimaging studies: Statistical power analyses. *J Neurosci Methods*. 2002;118(2):115-128. doi:10.1016/S0165-0270(02)00121-8

30. Glass G V. 9: Integrating Findings: The Meta-Analysis of Research. *Review of Research in Education*. 1977;5(1). doi:10.3102/0091732X005001351

31. Cohen J. *Statistical Power Analysis for the Behavioral Sciences*. 2nd ed. Lawrence Erlbaum Associates; 1988.

32. Benjamini Y, Hochberg Y. Controlling the False Discovery Rate: A Practical and Powerful Approach to Multiple Testing. *J R Stat Soc Series B Stat Methodol*. 1995;57(1). doi:10.1111/j.2517-6161.1995.tb02031.x

33. R Core Team. R: A language and environment for statistical computing. *R Foundation for Statistical Computing*. Preprint posted online 2023.

34. Jenkinson M, Beckmann CF, Behrens TEJ, Woolrich MW, Smith SM. FSL. *Neuroimage*. 2012;62(2):782-790. doi:10.1016/J.NEUROIMAGE.2011.09.015

35. Beckmann CF, DeLuca M, Devlin JT, Smith SM. Investigations into resting-state connectivity using independent component analysis. *Philos Trans R Soc Lond B Biol Sci*. 2005;360(1457):1001-1013. doi:10.1098/RSTB.2005.1634

36. Smith SM, Fox PT, Miller KL, et al. Correspondence of the brain’s functional architecture during activation and rest. *Proc Natl Acad Sci U S A*. 2009;106(31):13040-13045. doi:10.1073/PNAS.0905267106
